# Supplementary material for: Extensive Structure Modification on Luteolin-Cinnamic Acid Conjugates Leading to BACE1 Inhibitors with Optimal Pharmacological Properties
Source: Molecules. 2019 Dec 26;25(1):102. doi: 10.3390/molecules25010102 (PMC6982702; doi:10.3390/molecules25010102)
Supplement: Supplementary file 1 [file molecules-25-00102-s001.pdf]

molecules

MDPI

Supplementary Materials

Extensive Structure Modification on Luteolin-Cinnamic Acid Conjugates Leading to BACE1 Inhibitors with Optimal Pharmacological Properties

De-Yang Sun [1], Chen Cheng [2], Katrin Moschke [3,4], Jian Huang [2] and Wei-Shuo Fang [1,*]

1. State Key Laboratory of Bioactive Substances and Functions of Natural Medicines, Institute of Materia Medica, Chinese Academy of Medical Sciences and Peking Union Medical College , 2A Nan Wei Road, Beijing 100050, China; sdy887@126.com
2. College of Life Science, Wuhan University, Wuhan, Hubei 430072, China; cccc@whu.edu.cn (C.C.); jianhuang@whu.edu.cn (J.H.)
3. German Center for Neurodegenerative Diseases (DZNE) and Munich Cluster for Systems Neurology (SyNergy), Technical University of Munich, Feodor-Lynen-Strasse 17, 81377 Munich; katrin.moschke@dzne.de
4. Neuroproteomics, Klinikum rechts der Isar, Technical University of Munich, 81675 Munich, Germany

* Correspondence: wfang@imm.ac.cn; Tel.: +86-10-6316-5229

Academic Editors: Kamil Kuca, Tanos Celmar Costa Franca, Teodorico C. Ramalho
Received: 21 November 2019; Accepted: 23 December 2019; Published: date

Abstract: BACE1 inhibitory conjugates derived from two natural products luteolin (**1**) and *p*-hydroxy-cinnamic acid (**2**), are subjected to systematic structure modifications, including various positions in luteolin segment for conjugation, different linkers (length, bond variation), as well as various substitutions in cinnamic acid segment (various substituents on benzene, and replacement of benzene by heteroaromatics and cycloalkane). Optimal conjugates such as **7c** and **7k** are chosen on the basis of a series of bioassay data for further investigation.

Keywords: beta-secretase; BACE1 inhibitor; flavonoid; luteolin; cinnamic acid

Supplementary Materials:

1. Chemical synthesis

Table S1. The NMR data and MS data of compounds shown in the text.

Compd.	^{1}H-NMR (and ^{13}C-NMR)	MS or HR-MS
**16**	^{1}H-NMR (500 MHz, Acetone-d6) δ 12.70 (1H, s, 5-OH), 10.01 (1H, s, 3-OH), 7.84 (2H, m, H-5′,6′), , 7.51-7.35 (15H, m, -CH2Ph), 7.17 (1H, d, J = 8.8 Hz, H-2′), 6.67 (1H, d, J = 2.1 Hz, H-8), 6.38 (1H, d, J = 2.1 Hz, H-6), 5.28 – 5.19 (6H, m, -CH2Ph).	MS (ESI) *m/z:* 573.2 (M + H)$^+$
**17**	^{1}H-NMR (500 MHz, Acetone-d_6) δ 12.71 (1H, s, 5-OH), 7.88-7.85 (2H, m, H-5′,6′), , 7.51-7.35 (15H, m, -CH$_2$Ph), 7.17 (1H, d, *J* = 8.8 Hz, H-2′), 6.68 (1H, d, *J* = 2.1 Hz, H-8), 6.38 (1H, d, *J* = 2.1 Hz, H-6), 5.28 – 5.19 (6H, m, -CH$_2$Ph), 4.20 (2H, m, -OCH$_2$-), 3.85 (2H, m, -OCH$_2$-), 0.79 (9H, s, -C(CH$_3$)$_3$), -0.04 (6H, s, -SiCH$_3$).	MS (ESI) *m/z:* 793.2 (M + H)$^+$
**18**	^{1}H-NMR (400 MHz, DMSO-d_6) δ 12.69 (1H, s, 5-OH), 10.77 (1H,	MS (ESI) *m/z:*

	s, 7-OH), 9.70 (1H, s, 3′-OH), 9.25 (1H, s, 4′-OH), 7.56 (1H, dd, *J* = 8.5, 2.3 Hz, H-6′), 7.49 (1H, d, *J* = 2.2 Hz, H-2′), 6.82 (1H, d, *J* = 8.5 Hz, H- 5′), 6.35 (1H, d, *J* = 2.0 Hz, H-6), 6.14 (1H, d, *J* = 2.1 Hz, H-8), 4.20 (2H, m, $-OCH_2-$), 3.85 (2H, m, $-OCH_2-$), 0.76 (9H, s, $-C(CH_3)_3$), -0.07 (6H, s, $-SiCH_3$).	461.2 $(M + H)^+$,483.1 $(M + Na)^+$
**19**	^{1}H-NMR (400 MHz, Acetone-*d*$_6$) δ 12.71 (1H, s, 5-OH), 8.00 (1H, d, *J* = 2.2 Hz, H-2′), 7.93 (1H, m, H-6′), 7.29 (1H, d, *J* = 8.7 Hz, H-5′), 6.72 (1H, d, *J* = 2.2, H-6), 6.40 (1H, d, *J* = 2.2, H-8), 5.30 (6H, m, $-OCH_2O-$), 4.24 (2H, m, $-OCH_2-$), 3.93 (2H, m, $-OCH_2-$), 3.54 – 3.45 (9H, s, $-CH_3$), 0.82 (9H, s, $-C(CH_3)_3$), -0.02 (6H, s, $-SiCH_3$).	MS (ESI) *m/z:* 591.2 $(M + H)^+$.
**20**	^{1}H-NMR (500 MHz, DMSO-*d*$_6$) *δ* 12.95 (1H, s, 5-OH), 10.39 (1H, s, 4′-OH), 7.54 (1H, s, H-2′), 7.52 (1H, m, H-6′), 7.41 (1H, t, *J* = 8.0 Hz, H-5′), 7.22 (1H, dd, *J* = 8.1, 1.6 Hz, H-4′)6.86 (1H, s, H-3), 6.82 (1H, d, *J* = 1.5 Hz, H-8), 6.44 (1H, d, *J* = 1.5 Hz, H-6), 5.32 (2H, s, $-OCH_2O-$), 3.42 (3H, s, $-CH_3$).	MS (ESI) *m/z:* 331.2$(M + H)^+$
**21**	^{1}H-NMR (400 MHz, Acetone-*d*$_6$) δ 12.67 (1H, s, 5-OH), 8.03 (1H, d, *J* = 2.2 Hz, H-2′), 7.88 (1H, dd, *J* = 8.7, 2.2 Hz, H-6′), 7.57 – 7.45 (3H, m, 4″-OH and H-3″,5″), 7.26 (1H, d, *J* = 8.7 Hz, H-5′), 6.97 – 6.89 (3H, m, -<u>CH</u>=CHCO- and H-2″, 6″), 6.73 (1H, d, *J* = 1.8 Hz, H-6), 6.42 (1H, d, *J* = 2.1 Hz, H-8), 6.22 (1H, d, *J* = 16.0 Hz, -CH=<u>CH</u>CO-), 5.36 – 5.20 (6H, s, $-OCH_2O-$), 4.53 – 4.43 (4H, m, 2×$-OCH_2-$), 3.54 – 3.41 (9H, s, $-CH_3$) , 2.29 (3H, s, $-OCH_3$).	MS (ESI) *m/z:* 667.2 $(M + H)^+$
**22**	^{1}H-NMR (400 MHz, Acetone-*d*$_6$) δ 12.67 (1H, s, 5-OH), 8.03 (1H, d, *J* = 2.2 Hz, H-2′), 7.88 (1H, dd, *J* = 8.7, 2.2 Hz, H-6′), 7.57 – 7.45 (3H, m, 4″-OH and H-3″,5″), 7.26 (1H, d, *J* = 8.7 Hz, H-5′), 6.97 – 6.89 (3H, m, -<u>CH</u>=CHCO- and H-2″, 6″), 6.73 (1H, d, *J* = 1.8 Hz, H-6), 6.42 (1H, d, *J* = 2.1 Hz, H-8), 6.22 (1H, d, *J* = 16.0 Hz, -CH=<u>CH</u>CO-), 5.36 – 5.20 (6H, s, $-OCH_2O-$), 4.53 – 4.43 (4H, m, 2×$-OCH_2-$), 3.54 – 3.41 (9H, s, $-CH_3$).	MS (ESI) *m/z:* 625.2 $(M + H)^+$
**4j**	^{1}H-NMR (400 MHz, DMSO-*d*$_6$) δ 12.90 (1H, s, 5-OH), 10.93 (1H, s, 3′-OH), 10.84 (1H, s, 4′-OH), 10.03 (1H, s, 4′-OH), 9.99 (1H, s, 7-OH), 8.02 (1H, d, *J* = 2.2 Hz, H-2′), 7.88 (1H, dd, *J* = 8.7, 2.2 Hz, H-6′), 7.57 – 7.45 (3H, m, 4″-OH and H-3″,5″), 7.24 (1H, d, *J* = 8.7 Hz, H-5′), 6.97 – 6.89 (3H, m, -<u>CH</u>=CHCO- and H-2″, 6″), 6.75 (1H, d, *J* = 1.8 Hz, H-6), 6.40 (1H, d, *J* = 2.1 Hz, H-8), 6.19 (1H, d, *J* = 16.0 Hz, -CH=<u>CH</u>CO-), 4.53 – 4.43 (4H, m, 2×$-OCH_2-$). ^{13}C-NMR (150MHz, DMSO-*d*$_6$) δ 182.20, 167.02, 164.73, 163.66, 161.97, 161.89, 160.29, 157.76, 145.40, 130.78, 128.74, 125.43, 123.35, 116.15, 115.50, 114.44, 109.99, 104.19, 103.96, 100.88, 99.31, 98.66, 94.47, 68.07, 66.10.	HR-MS (ESI) calcd for $C_{26}H_{20}O_{10}$: 492.10565, found$[M + H]^+$: 493.10712.
**23**	^{1}H-NMR(400MHz, DMSO-*d*$_6$) δ 6.20 (1H, d, *J* = 0.8Hz, H-6), 6.52 (1H, d, *J* = 1.2Hz, H-8), 6.89 (1H, s, H-3), 7.23 (1H, d, *J* = 8.4Hz, H-6′), 7.40-7.61 (10H, m, H of diphenylmethane), 7.71 (1H, d, *J* = 8.0Hz, H-5′), 7.79 (1H, s, H-2′).	MS (ESI) m/z: 451.1 $(M + H)^+$.
**24**	^{1}H-NMR (400 MHz, Acetone-*d*$_6$) δ 12.85 (1H, s, 5-OH), 7.68 (1H, m, H-6′), 7.66 (1H, d, *J* = 1.7 Hz, H-2′), 7.62–7.58 (4H, m, H-*o* of diphenylmethane), 7.46–7.41 (6H, m, H-*m, p* of diphenylmethane), 7.14 (1H, d, *J* = 8.2 Hz, H-5′), 6.69 (1H, d, *J* = 1.9 Hz, H-8), 6.69 (1H, s, H-3), 6.30 (1H, d, *J* = 2.2 Hz, H-6), 4.15 (2H, t, *J* = 6.6 Hz, $-OCH_2-$), 3.71 (2H, t, *J* = 6.2 Hz, $-CH_2OTBS$), 1.92 – 1.62 (4H, m, $-CH_2-$), 0.88 (9H, s, $-C(CH_3)_3$), 0.05 (6H, s, $-Si(CH_3)_2$).	MS (ESI) *m/z:* 637.3 $(M + H)^+$

**25a**	^{1}H-NMR (400 MHz, Acetone-*d*$_6$) δ 7.71-7.67 (2H, m, H-2',H-6'), 7.61-7.59 (4H, m, H-*o* of diphenylmethane), 7.45-7.43 (6H, m, H-*m, p* of diphenylmethane), 7.15 (1H, d, *J* = 8.0 Hz, H-5'), 6.72 (1H, s, H-8), 6.70 (1H, s, H-3), 6.30 (1H, s, H-6), 4.23 (2H, m, TsOCH$_2$-), 3.71 (2H, m, -CH$_2$OH), 1.98 (2H, m, -CH$_2$-).	MS (ESI) *m/z:* 509.2 (M + H)$^+$
**25c**	^{1}H-NMR (400 MHz, Acetone-*d*$_6$) δ 12.85 (1H, s, 5-OH), 7.67 (2H, m, H-2',H-6'), 7.62 – 7.57 (4H, m, H-*o* of diphenylmethane), 7.45-7.43 (6H, m, H-*m, p* of diphenylmethane), 7.13 (1H, d, *J* = 8.2 Hz, H-5'), 6.69 (2H, m, H-3, H-8), 6.29 (1H, s, H-6), 4.11 (2H, t, *J* = 6.5 Hz, -OCH$_2$-), 3.55 (2H, s, -CH$_2$OH), 1.83-1.32 (6H, m, -CH$_2$-).	MS (ESI) *m/z:* 537.2 (M + H)$^+$
**25d**	^{1}H-NMR (400 MHz, Acetone-d$_6$) δ 12.85 (1H, s, 5-OH), 7.66 (2H, m, H-2', H-6'), 7.61-7.58 (4H, m, H-*o* of diphenylmethane), 7.45-7.43 (6H, m, H-*m, p* of diphenylmethane), 7.12 (1H, d, *J* = 8.2 Hz, H-5'), 6.68 (2H, m, H-3, H-8), 6.28 (1H, s, H-6), 4.09 (2H, t, *J* = 5.9 Hz, -OCH$_2$-), 3.53 (2H, s, -CH$_2$OH), 1.83-1.44 (8H, m, -CH$_2$-).	MS (ESI) *m/z:* 551.2 (M + H)$^+$
**25e**	^{1}H-NMR (400 MHz, Acetone-d$_6$) δ 7.57 (1H, s, H-2'), 7.38 (4H, m, H-*o* of diphenylmethane), 7.25 (6H, m, H-*m,p* of diphenylmethane), 7.24 (1H, s, H-6'), 6.96 (1H, s, H-5'), 6.50 (1H, s, H-8), 6.43 (1H, s, H-3), 6.32 (1H, s, H-6), 3.99 (2H, s, -OCH$_2$-), 3.63 (2H, s, -CH$_2$OH) , 1.79-1.36 (8H, m, -CH$_2$-).	MS (ESI) *m/z:* 579.2 (M + H)$^+$, 601.2 (M + Na)$^+$
**25f**	^{1}H-NMR (400 MHz, Acetone-*d*$_6$) δ 7.64 – 7.54 (4H, m, H-*o* of diphenylmethane), 7.43 (1H, dd, *J* = 8.3, 1.9 Hz, H-6′), 7.40 -7.36 (7H, m, H-*m, p* of diphenylmethane and H-2′), 6.96 (1H, d, *J* = 8.3 Hz, H-5'), 6.50 (1H, s, H-3), 6.43 (1H, d, *J* = 2.2 Hz, H-8), 6.32 (1H, d, *J* = 2.2 Hz, H-6), 3.99 (2H, t, *J* = 6.4 Hz, -OCH$_2$-), 3.63 (2H, t, *J* = 6.6 Hz, -CH$_2$OH), 1.78 -1.31 (12H, m, -CH2-).	MS (ESI) *m/z:* 607.2 (M + H)$^+$
**25b**	^{1}H-NMR (400 MHz, Acetone-*d*$_6$) δ 12.85 (1H, s, 5-OH), 7.68 (1H, dd, *J* = 8.2, 1.9 Hz, H-6′), 7.66 (1H, d, *J* = 1.7 Hz, H-2′), 7.62-7.58 (4H, m, H-*o* of diphenylmethane), 7.46-7.41 (6H, m, H-*m, p* of diphenylmethane), 7.14 (1H, d, *J* = 8.2 Hz, H-5′), 6.70 (1H, d, *J* = 2.2 Hz, H-8), 6.69 (1H, s, H-3), 6.29 (1H, d, *J* = 2.2 Hz, H-6), 4.14 (2H, t, *J* = 6.6 Hz, -OCH$_2$-), 3.60 (2H, t, *J* = 6.6 Hz, -CH$_2$OH), 1.92 – 1.63 (4H, m, -CH$_2$-).	MS (ESI) *m/z:* 523.2 (M + H)$^+$
**26a**	^{1}H-NMR (400 MHz, Acetone-d$_6$) δ 12.87 (1H, s, 5-OH), 7.74 – 7.63 (5H, m, H-2″,6″ and H-2′, H-6′, -<u>CH</u>=CHCO-), 7.62 – 7.57 (4H, m, H-*o* of diphenylmethane), 7.48 – 7.40 (6H, m, H-*m, p* of diphenylmethane), 7.15 (2H, d, *J* = 9.0 Hz, H-3″,5″), 7.13 (1H, d, *J* = 8.0 Hz, H-5′), 6.73 (1H, d, *J* = 2.2 Hz, H-8), 6.69 (1H, s, H-3), 6.52 (1H, d, *J* = 16.0 Hz, -CH=<u>CH</u>CO-), 6.33 (1H, d, *J* = 2.3 Hz, H-6), 4.37 (3H, t, *J* = 6.3 Hz, -OCH$_2$-), 4.28 (2H, t, *J* = 6.2 Hz, -CH$_2$OH), 2.23 (3H, s, -CH$_3$), 2.20 (2H, m, -CH$_2$-).	MS (ESI) *m/z:* 697.2 (M + H)$^+$
**26b**	^{1}H-NMR (400 MHz, CDCl$_3$) δ 7.65 (1H, d, *J* = 16.0 Hz, -<u>CH</u>=CHCO-), 7.60 – 7.57 (4H, m, H-*o* of diphenylmethane), 7.51 (2H, d, *J* = 8.6 Hz, H-2″,6″), 7.45 (1H, dd, *J* = 8.3, 1.7 Hz, H-6′), 7.42 –7.38 (7H, m, H-*m, p* of diphenylmethane and H-2′), 7.10 (2H, d, *J* = 8.6 Hz, H-3″,5″), 6.99 (1H, d, *J* = 8.3 Hz, H-5′), 6.51 (1H, s, H-3), 6.46 (1H, d, *J* = 2.2 Hz, H-8), 6.38 (1H, d, *J* = 12.8 Hz, -CH=<u>CH</u>CO-), 6.35 (1H, s, H-6), 4.30 (t, *J* = 5.7 Hz, -OCH$_2$-), 4.10 (2H, t, *J* = 5.7 Hz, -CH$_2$OH), 2.31 (3H, s, -CH$_3$), 1.94 (4H, m, -CH$_2$-).	MS (ESI) *m/z:* 711.2 (M + H)$^+$

**26c**	^{1}H-NMR (400 MHz, Acetone-d_6) δ 12.85 (1H, s, 5-OH), 7.69- 7.66 (5H, m, H-2″,6″ and H-2′, H-6′, -CH=CHCO-), 7.61-7.58 (4H, m, H-*o* of diphenylmethane), 7.45-7.43 (6H, m, H-*m,p* of diphenylmethane), 7.16-7.12 (3H, m, H-3″,5″ and H- 5′), 6.70 (1H, s, H-8), 6.68 (1H, s, H-3), 6.50 (1H, d, *J*=16.0Hz, -CH=CHCO-), 6.30 (1H, s, H-6), 4.18 (2H, m, -OCH_2-) , 4.15 (2H, m, -CH_2OH), 2.23 (3H, s, -CH_3), 1.88-1.58 (6H, m, -CH_2-).	MS (ESI) *m/z:* 725.2 (M + H)$^+$, 747.2 (M + Na)$^+$
**26d**	^{1}H-NMR (400 MHz,$CDCl_3$) δ 7.64 (1H, d, *J* = 16.0 Hz, -CH=CHCO-), 7.60–7.55 (4H, m, H-*o* of diphenylmethane), 7.51 (2H, d, *J* = 8.6 Hz, H-2″,6″), 7.45 (1H, dd, *J* = 8.3, 1.8 Hz, H-6′), 7.38 (7H, m, H-*m,p* of diphenylmethane and H-2′), 7.10 (2H, d, *J* = 8.6 Hz, H-3″,5″), 6.98 (1H, d, *J* = 8.3 Hz, H-5′), 6.50 (1H, s, H-3), 6.44 (1H, d, *J* = 2.2 Hz, H-8), 6.38 (1H, d, *J* = 16.0 Hz, -CH=CHCO-), 6.33 (1H, d, *J* = 2.2 Hz, H-6), 4.22 (2H, t, *J* = 6.6 Hz, -OCH_2-), 4.02 (2H, t, J = 6.4 Hz, -CH_2OH), 2.29 (3H, s, -CH_3), 1.88 – 1.46 (8H, m, -CH_2-).	MS (ESI) *m/z:* 739.2 (M + H)$^+$
**26e**	^{1}H-NMR (400 MHz, Acetone-d_6) δ 12.85 (1H, s, 5-OH), 7.69-7.64 (5H, m, H-2″,6″ and H-2′,H-6′, -CH=CHCO-), 7.61-7.58 (4H, m, H-*o* of diphenylmethane), 7.45- 7.42 (6H, m, H-*m, p* of diphenylmethane), 7.13(3H,m, H-3″,5″ and H-5′), 6.67(2H, m, H-3, H-8), 6.50 (1H, d, *J*=16.0Hz, -CH=CHCO-), 6.28 (1H, s, H-6), 4.14(2H, m, -OCH_2-), 4.10 (2H, m, -CH_2OH), 2.34 (3H, s, -CH_3), 1.80-1.39(12H, m, -CH_2-).	MS (ESI) *m/z:* 767.2 (M + H)$^+$
**26f**	^{1}H-NMR (400 MHz, Acetone-d6) δ 12.85 (1H, s, 5-OH), 7.69 (5H, m, H-2″,6″ and H-2′,H-6′, -CH=CHCO-), 7.63 – 7.58 (4H, m, H-o of diphenylmethane), 7.47 – 7.41 (6H, m, H-m, p of diphenylmethane), 7.14 (3H,m, H-3″,5″ and H-5′), 6.68 (2H, m, H-3, H-8), 6.50 (1H, d, J=16.0Hz, -CH=CHCO-), 6.28 (1H, s, H-6), 4.15 (2H, m, -OCH2-), 4.09 (2H, m, -CH2OH), 2.23 (3H, s, -CH3), 1.78-1.21 (16H, m, -CH2-).	MS (ESI) *m/z:* 795.2 (M + H)$^+$, 817.2 (M + Na)$^+$
**5a**	^{1}H-NMR (400 MHz, DMSO-d6) δ 12.97 (1H, s, 5-OH), 7.58 (1H, d, J = 14.7 Hz, -CH=CHCO-), 7.55 (2H, d, J = 8.4 Hz, H-2″,6″), 7.47 –7.42 (2H, m, H-2′,H-6′), 6.89 (1H, d, J = 8.0 Hz, H-5′), 6.78 (2H, d, J = 8.6 Hz, H-3″,5″), 6.76 (1H, s, H-8), 6.73 (1H, s, H-3), 6.40 (1H, d, J = 16.1 Hz, -CH=CHCO-), 6.39 (1H, d, J = 1.9 Hz, H-6), 4.29 (2H, t, J = 6.1 Hz, -OCH2-), 4.23 (2H, t, J = 5.9 Hz, -CH2O-), 2.18 – 2.10 (2H, m, -CH2-).13C-NMR (151 MHz, DMSO-d6) δ 182.23, 167.02, 164.69, 164.67, 161.63, 160.27, 157.62, 150.26, 146.19, 145.26, 130.75, 125.48, 121.83, 119.51, 116.37, 116.17, 114.46, 113.97, 105.12, 103.48, 98.76, 93.45, 65.80, 61.06, 28.40.	HR-MS (ESI) calcd for $C_{27}H_{22}O_9$: 490.12638, found[M + H]$^+$: 491.13333.
**5b**	^{1}H-NMR (400 MHz,Acetone-d_6) δ 12.98 (1H, s, 5-OH), 8.89 (1H, s, 4″-OH), 8.88 (1H, s, 4′-OH), 8.44 (1H, s, 3′-OH), 7.60 (1H, d, *J* = 15.9 Hz, -CH=CHCO-), 7.54 – 7.47 (4H, m, H-2″,6″ and H-2′, H-5′), 7.01 (1H, d, *J* = 8.3 Hz, H-5′), 6.87 (2H, d, *J* = 8.6 Hz, H-3″,5″), 6.69 (1H, d, *J* = 2.1 Hz, H-8), 6.60 (1H, s, H-3), 6.34 (d, *J* = 2.0 Hz, H-6), 6.33 (1H, d, *J* = 15.9 Hz, -CH=CHCO-), 4.25 (4H, m, -OCH_2-), 2.02 – 1.87 (4H, m, -CH_2-). ^{13}C-NMR (150 MHz, Acetone-d_6) δ 182.21, 167.04, 164.85, 164.64, 161.62, 160.23, 157.63, 150.24, 146.19, 145.08, 130.69, 130.51, 125.47, 121.85, 119.50, 116.37, 116.15, 114.56, 113.96, 105.05, 103.48, 98.74, 93.40, 68.44, 63.86, 25.60, 25.34.	HR-MS (ESI) calcd for $C_{28}H_{24}O_9$: 504.14203, found[M + H]$^+$: 505.14862.

**5c**	^{1}H-NMR (400 MHz, DMSO-d_6) δ 7.56 (3H, m, -CH=CHCO- and H-2″,6″), 7.43 (2H, m, H-2′, H-5′), 6.85 (1H, d, *J* = 7.9 Hz, H-5′), 6.79 (2H, d, *J* = 6.6 Hz, H-3″,5″), 6.72 (1H, s, H-3), 6.69 (1H, s, H-8), 6.39 (1H, d, *J* = 15.9, -CH=CHCO-), 6.35 (1H, s, H-6), 4.15 (4H, m, $-OCH_2-$), 1.79-1.52 (6H, m, $-CH_2-$). ^{13}C-NMR (151 MHz, DMSO-d_6) δ 182.21, 167.10, 164.92, 164.64, 161.59, 157.65, 150.26, 146.19, 145.08, 132.81, 130.71, 125.48, 121.83, 119.50, 116.39, 115.31, 114.61, 113.93, 106.16, 103.45, 98.73, 93.46, 93.38, 68.74, 64.07, 28.45, 28.38, 27.85, 21.49.	HR-MS (ESI) calcd for $C_{29}H_{26}O_9$: 518.15768, found $[M + H]^+$: 519.16473.
**5d**	^{1}H-NMR (400 MHz,Acetone-d_6) δ 12.94 (1H, s, 5-OH), 8.92 (1H, s, 4″-OH), 8.84 (1H, s, 4′-OH), 8.47 (1H, s, 3′-OH), 7.57 (1H, d, *J*=16.0Hz, -CH=CHCO-), 7.51-7.49 (3H, m, 2H, s, H-2″,6″ and H-2′), 7.46 (1H, dd, *J* = 8.3, 2.2 Hz, H-6′), 6.97 (1H, d, *J* = 8.4 Hz, H-5′), 6.85 (2H, d, *J* = 8.6 Hz, H-3″,5″), 6.65 (1H, d, *J* = 2.1 Hz, H-8), 6.58 (1H, s, H-3), 6.30(1H, d, *J*=16.0Hz, -CH=CHCO-), 6.29 (1H, s, H-6), 4.15 (4H, q, *J* = 6.5 Hz, $-OCH_2-$), 1.90 – 1.46 (8H, m, $-CH_2-$). ^{13}C-NMR (150 MHz, Acetone-d_6) δ 182.21, 167.09, 164.93, 164.62, 161.60, 160.22, 157.63, 150.24, 146.18, 145.04, 130.71, 130.07, 125.48, 121.85, 119.50, 116.37, 116.15, 114.62, 113.96, 105.00, 103.46, 98.71, 93.36, 68.78, 64.10, 28.71, 28.64, 25.56, 25.51.	HR-MS (ESI) calcd for $C_{30}H_{28}O_9$: 532.17333, found$[M + H]^+$: 533.17999.
**5e**	^{1}H-NMR (400 MHz,DMSO-d_6) δ 12.96 (1H, s, 5-OH), 9.99 (1H, s, 4″-OH), 9.97 (1H, s, 4′-OH), 9.36 (1H, s, 3′-OH), 7.53 (1H, d, *J*=16.0Hz, -CH=CHCO-), 7.54 (2H, d, *J* = 8.7 Hz, H-2″,6″), 7.45 (1H, m, H-6′), 7.43 (1H, d, *J* = 2.2 Hz, H-2′), 6.90 (1H, d, *J* = 8.2 Hz, H-5′), 6.78 (2H, d, *J* = 8.6 Hz, H-3″,5″), 6.72 (1H, s, H-3), 6.72 (1H, d, *J* = 2.3 Hz, 1H, s, H-8), 6.38 (1H, d, *J* = 16.0 Hz, -CH=CHCO-), 6.35 (1H, d, *J* = 2.2 Hz, H-6), 4.10 (4H, m, $-OCH_2-$), 1.80 – 1.29 (12H, m, $-CH_2-$).^{13}C-NMR (150 MHz, DMSO-d_6) δ 182.21, 167.08, 164.95, 164.62, 161.60, 160.22, 157.64, 150.24, 146.18, 145.03, 133.13, 130.72, 130.03, 129.01, 125.50, 121.86, 119.50, 116.37, 116.16, 114.64, 113.97, 105.00, 103.46, 98.71, 93.34, 68.85, 64.14, 29.03, 29.02, 28.79, 28.69, 25.81, 25.74.	HR-MS (ESI) calcd for $C_{32}H_{32}O_9$: 560.20463, found$[M + H]^+$: 561.20513.
**5f**	^{1}H-NMR (400 MHz, DMSO-d_6) δ 12.96 (1H, s, 5-OH), 9.99 (1H, s, 4″-OH), 9.98 (1H, s, 4′-OH), 9.37 (1H, s, 3′-OH), 7.58 – 7.52 (3H, m, -CH=CHCO- and H-2″,6″), 7.44 (2H, t, H-2′, H-6′), 6.90 (1H, d, *J* = 8.2 Hz, H-5′), 6.78 (2H, d, *J* = 8.6 Hz, H-3″,5″), 6.72 (1H, s, H-8), 6.72 (1H, s, H-3), 6.38 (1H, d, *J* = 16.0 Hz, -CH=CHCO-), 6.35 (1H, s, H-6), 4.10 (4H, m, $-OCH_2-$), 1.77 – 1.27 (16H, m, $-CH_2-$).^{13}C-NMR (150 MHz, DMSO-d_6) δ 182.21, 167.08, 164.95, 164.62, 161.59, 160.22, 157.64, 150.24, 146.18, 145.02, 130.72, 125.50, 121.85, 119.50, 116.37, 116.16, 114.64, 113.96, 104.99, 103.46, 98.72, 93.34, 68.85, 64.15, 29.33, 29.29, 29.08, 28.80, 28.69, 25.86, 25.79.	HR-MS (ESI) $[M + H]^+$ calcd for $C_{34}H_{36}O_9$: 588.23593, found$[M + H]^+$: 589.24146.
**27**	^{1}H-NMR (400MHz, $CDCl_3$) δ 3.99 (2H, t, *J* = 4.0Hz, $-OCH_2-$), 4.13 (2H, t, *J* = 4.0Hz, $-OCH_2-$), 6.34 (1H, d, *J* = 1.6Hz, H-6), 6.45 (1H, d, *J* = 1.6Hz, H-8), 6.50 (1H, s, H-3), 6.96 (1H, d, *J* = 8.0Hz, H-6′), 7.34-7.46 (8H, m, H of diphenylmethane, H-2′,H-5′), 7.54-7.63 (4H, m, H of diphenylmethane).	MS (ESI) m/z: 495.1 $(M + H)^+$.
**28a**	^{1}H-NMR (400MHz, $CDCl_3$) δ 7.74 (1H, d, *J*=16.0 Hz, -CH=CHCO-), 7.60-7.57 (4H, m, H-*o* of diphenylmethane), 7.45 (1H,dd, *J*=8.0Hz, 3.4 Hz, H-6'), 7.43-7.38 (10H, m, H-2' and H-3'',	MS (ESI) *m/z:* 625.2 $(M + H)^+$

	4'', 5'' and H-*m ,p* of diphenylmethane), 6.99 (1H, d, *J*=8.0 Hz, H-5'), 6.53 (1H, s, H-3), 6.51 (1H, d, *J*=3.4 Hz, H-8), 6.49 (1H, d, *J*=16.0 Hz, -CH=CHCO-), 6.41 (1H, d, *J*=3.4 Hz, H-6), 4.59 (2H, t, *J* = 4.4 Hz, -OCH$_2$-), 4.32 (2H, t, *J* = 4.6 Hz, -OCH$_2$-).	
**28b**	.^{1}H-NMR (500 MHz, DMSO-*d*$_6$) *δ* 12.90 (1H, s, 5-OH), 7.98 (1H, d, *J* = 7.6 Hz, H-6''), 7.85(1H, s, H-2'), 7.78 (1H, d, *J* = 8.2 Hz, H-6'), 7.67(1H, d, *J*= 16.0 Hz, -CH=CHCO-), 7.61-7.60(4H, m, H-*o* of diphenylmethane), 7.52-7.51 (7H, m, H-4'' and H-*m, p* of diphenylmethane), 7.34 (1H, t, *J*=7.5 Hz, H-5''), 7.29 (1H, d, *J*=8.3 Hz, H-5'), 7.25(1H, d, *J*=8.0 Hz, H-3'') 7.13, 7.01 (1H, s, H-3), 6.93 (1H, s, H-8), 6.78 (1H, d, *J*=16.0 Hz, -CH=CHCO-), 6.47 (1H, s, H-6), 4.57 (2H, s, -OCH$_2$-), 4.45 (2H, s, -OCH$_2$-), 2.36 (3H, s, -CH$_3$).	MS (ESI) *m/z:* 683.2 (M + H)$^+$
**28c**	^{1}H-NMR (400 MHz, DMSO-*d*$_6$) *δ* 12.83 (1H, s, 5-OH), 7.80 (1H, s, H-2''), 7.72 (1H, d, *J* = 8.4 Hz, H-6'), 7.65 (1H, d, *J* = 15.9 Hz, -CH=CHCO-), 7.59 (1H, d, *J* = 7.4 Hz, H-6''), 7.55-7.52 (4H, m, H-*o* of diphenylmethane), 7.46-7.44 (6H, m, H-*m, p* of diphenylmethane), 7.40 (1H, t, *J* = 8.0 Hz, H-5''), 7.22 (1H, d, *J* = 8.3 Hz, H-5'), 7.16 (1H, d, *J* = 8.1 Hz, H-4''), 6.94 (1H, s, H-3), 6.87 (1H, s, H-8), 6.71 (1H, d, *J* = 16.0 Hz, -CH=CHCO-), 6.41 (1H, s, H-6), 4.50 (2H, s, -OCH$_2$-), 4.40 (2H, s, -OCH$_2$-), 2.24 (3H, s, -CH$_3$).	MS (ESI) *m/z:* 683.2 (M + H)$^+$
**28d**	^{1}H-NMR (400 MHz, Acetone-*d*$_6$) *δ* 7.69-7.65 (3H, m, -CH=CHCO- and H-2' and H-2''), 7.62-7.59 (6H, m, H-6' and H-6'' and H-*o* of diphenylmethane), 7.49-7.46 (6H, m, H-*m, p* of diphenylmethane), 7.30 (1H, d, *J* = 8.1 Hz, H-5''), 7.17 (1H, d, *J* = 8.0 Hz, H-5'), 6.80 (1H, s, H-3), 6.74 (1H, s, H-8), 6.60 (1H, d, *J* = 16.1 Hz, CH=CHCO-), 6.40 (1H, s, H-6), 4.59 (2H, s, -OCH$_2$-), 4.48 (2H, s, -OCH$_2$-), 2.28 (6H, s, -CH$_3$).	MS (ESI) *m/z:* 741.2 (M + H)$^+$
**28e**	^{1}H-NMR (400 MHz, CDCl$_3$) *δ* 7.69 (1H, d, *J* = 16.0 Hz, -CH=CHCO-), 7.61-7.55 (4H, m, H-*o* of diphenylmethane), 7.51 (2H, dd, *J* = 8.7, 5.4 Hz, H-3'', 5''), 7.45 (1H, dd, *J* = 8.4, 1.4 Hz, H-6'), 7.40-7.38 (7H, m, H-*m, p* of diphenylmethane and H-2'), 7.06 (2H, t, *J* = 8.6 Hz, H-2'',6''), 6.99 (1H, d, *J* = 8.2 Hz, H-5'), 6.53 (1H, s, H-3), 6.50 (1H, d, *J* = 2.1 Hz, H-8), 6.40 (1H, d, *J* = 16.0 Hz, -CH=CHCO-), 6.40 (1H, d, *J* = 2.0 Hz, H-6), 4.59 (2H, t, *J*=4.5Hz, -OCH$_2$-), 4.34 (2H, t, *J*=4.4 Hz, -OCH$_2$-).	MS (ESI) *m/z:* 643.2 (M + H)$^+$
**28f**	^{1}H-NMR (400 MHz, Acetone-*d*$_6$) *δ* 12.91 (1H, s, 5-OH), 7.72-7.68 (5H, m, H-2'', 6'' and H-2', 6' and -CH=CHCO-), 7.64-7.62 (4H, m, H-*o* of diphenylmethane), 7.48-7.43(8H, m, H-*m,p* of diphenylmethane, and H-3'', 5''), 7.17 (1H, d, *J* = 8.0 Hz, H-5'), 6.80 (1H, s, H-3), 6.73 (1H, s, H-8), 6.62 (1H, d, *J* = 16.1 Hz, -CH=CHCO-), 6.39 (1H, s, H-6), 4.59 (2H, s, -OCH$_2$-), 4.48 (2H, s, -OCH$_2$-).	MS (ESI) *m/z:* 659.1 (M + H)$^+$
**28g**	^{1}H-NMR (400 MHz, Acetone-*d*$_6$) *δ* 12.92 (1H, s, 5-OH), 8.26 (2H, d, *J* = 8.5 Hz, H-3'', 5''), 7.99 (2H, d, *J* = 9.3 Hz, H-2'', 6''), 7.82 (1H, d, *J* = 16.1 Hz, -CH=CHCO-), 7.71 (1H, d, *J* = 8.0 Hz, H-6'), 7.70 (1H, s, H-2'), 7.66-7.62 (4H, m, H-*o* of diphenylmethane), 7.46 (6H, m, H-*m, p* of diphenylmethane), 7.18 (1H, d, *J* = 8.0 Hz, H-5'), 6.81(1H, s, H-3), 6.82 (1H, d, *J* = 16.1 Hz, -CH=CHCO-), 6.74 (1H, s, H-8), 6.40 (1H, s, H-6), 4.63 (2H, s, -OCH$_2$-), 4.50 (2H, s, -OCH$_2$-).	MS (ESI) *m/z:* 670.2 (M + H)$^+$

**28h**	^{1}H-NMR (400 MHz, $CDCl_3$) δ 12.76 (1H, s, 5-OH), 7.66 (1H, d, *J* = 15.8 Hz, -<u>CH</u>=CHCO-), 7.60-7.54 (4H, m, H-*o* of diphenylmethane), 7.48-7.42 (4H, m, H-3'', 5'' and H-2', 6'), 7.42-7.38 (6H, m, H-*m, p* of diphenylmethane), 6.99 (1H, d, *J* = 8.2 Hz, H-5'), 6.77 (2H, d, *J* = 7.4 Hz, H-2'', 6''), 6.53 (1H, s, H-3), 6.51 (1H, d, *J* = 2.0 Hz, H-8), 6.40 (1H, s, *J* = 2.0 Hz, H-6), 6.28 (1H, d, *J* = 15.8 Hz, -CH=<u>CH</u>CO-), 4.57 (2H, t, *J*=3.4Hz, $-OCH_2-$), 4.31 (2H, t, *J*=3.4 Hz, $-OCH_2-$), 3.02 (6H, s, $-CH_3$).	MS (ESI) *m/z:* 668.2 $(M + H)^+$
**28i**	^{1}H-NMR (400 MHz, Acetone-d_6) δ 12.91 (1H, s, 5-OH), 7.71 (3H, m, *J* = 14.3 Hz, -<u>CH</u>=CHCO- and H-2', 6'), 7.63 (6H, m, H-2'', 6'' and H-*o* of diphenylmethane), 7.47 (6H, m, H-*m, p* of diphenylmethane), 7.17 (1H, d, *J* = 8.2 Hz, H-5'), 6.96 (2H, d, *J* = 7.5 Hz, H-3'', 5''), 6.81 (1H, s, H-3), 6.73 (1H, s, H-8), 6.44 (1H, d, *J* = 16.1 Hz, -CH=<u>CH</u>CO-), 6.39 (1H, s, H-6), 4.56 (2H, s, $-OCH_2-$), 4.47 (2H, s, $-OCH_2-$), 3.83 (3H, s, $-OCH_3$).	MS (ESI) *m/z:* 665.2 $(M + H)^+$, 677.2 $(M + Na)^+$
**28j**	^{1}H-NMR (400 MHz, Acetone-d_6) δ 12.91 (1H, s, 5-OH), 7.71-7.67 (3H, m, d, H-2', 6'and -<u>CH</u>=CHCO-), 7.63-7.59(6H, m, H-*o* of diphenylmethane and H-2'', 6''), 7.62 (8H, m, H-*p, m* of diphenylmethane, and H-3'', 5''), 7.46 (s, 12H), 7.16 (2H, d, *J* = 8.0 Hz, H-5'), 6.81 (1H, s, H-3), 6.72 (1H, s, H-6), 6.54 (1H, d, *J* = 16.0 Hz, -CH=<u>CH</u>CO-), 6.39 (1H, s, H-6), 4.58 (2H, s, $-OCH_2-$), 4.48 (2H, s, $-OCH_2-$), 1.31 (9H, s, $-CH_3$).	MS (ESI) *m/z:* 681.2 $(M + H)^+$
**28k**	^{1}H-NMR (400 MHz, Acetone-d_6) δ 12.92 (1H, s, 5-OH), 8.85 (1H, s, H-2''), 8.58 (1H, s, H-4''), 8.11 (1H, s, H-6''), 7.78-7.71 (3H, m, d, H-2', 6' and -<u>CH</u>=CHCO-), 7.62 (4H, m, H-*o* of diphenylmethane), 7.46 (6H, m, H-*p, m* of diphenylmethane), 7.43 (1H, m, H-5''), 7.17 (2H, d, *J* = 8.4 Hz, H-5'), 6.82 (1H, s, H-3), 6.74 (1H, s, H-6), 6.73 (1H, d, *J* = 16.0 Hz, -CH=<u>CH</u>CO-), 6.40 (1H, s, H-6), 4.61 (2H, s, $-OCH_2-$). 4.49 (2H, s, $-OCH_2-$).	MS (ESI) *m/z:* 626.2 $(M + H)^+$, 648.2 $(M + Na)^+$
**28l**	^{1}H-NMR (400 MHz, Acetone-d_6) δ 12.86 (1H, s, 5-OH), 7.92 (1H, s, H-2″), 7.65 (3H, m, d, H-2', 6'and -<u>CH</u>=CHCO-), 7.57 (5H, m, H-*o* of diphenylmethane and H-5″), 7.41 (6H, m, H-*p, m* of diphenylmethane), 7.11 (1H, d, *J* = 8.1 Hz, H-5′), 6.84 (1H, s, H-4″), 6.74 (1H, s, H-3), 6.68 (1H, s, H-8), 6.33 (1H, s, H-6), 6.28 (1H, d, *J* = 15.7 Hz, -CH=<u>CH</u>CO-), 4.50 (2H, s, $-OCH_2-$), 4.40 (2H, s, $-OCH_2-$).	MS (ESI) *m/z:* 615.2 $(M + H)^+$, 637.2 $(M + Na)^+$
**28m**	^{1}H-NMR (400 MHz, Acetone-d_6) δ 12.90 (1H, s, 5-OH), 7.70 (2H, m, d, H-2′,6′), 7.63 (4H, m, H-*o* of diphenylmethane), 7.47 (6H, m, H-*p, m* of diphenylmethane), 7.16 (1H, d, *J* = 8.2 Hz, H-5′), 6.90 (1H, dd, *J* = 15.5, 6.9 Hz, -<u>CH</u>=CHCO-), 6.78 (1H, s, H-3), 6.73 (1H, s, H-8), 6.36 (1H, s, H-6), 5.80 (1H, d, *J* = 15.8 Hz, -CH=<u>CH</u>CO-), 4.50 (2H, s, $-OCH_2-$), 4.43 (2H, s, $-OCH_2-$),2.52 – 2.45 (1H, m, -CH), 1.72 – 1.64 (4H, m, $-CH_2-$), 1.32 – 1.02 (6H, m, $-CH_2-$).	MS (ESI) *m/z:* 628.8 $(M - H)^-$
**28n**	^{1}H-NMR (400 MHz, Acetone-d_6) δ 12.90 (1H, s, 5-OH), 7.71 (2H, m, d, H-2′,6′), 7.63 (4H, m, H-*o* of diphenylmethane), 7.47 (6H, m, H-*p, m* of diphenylmethane), 7.17 (1H, d, *J* = 8.1 Hz, H-5′), 6.76 (1H, s, H-4″), 6.74 (1H, s, H-3), 6.35 (1H, s, H-8), 5.70 (1H, s, H-6), 4.45 (2H, m, $-OCH_2-$), 4.36 (2H, m, $-OCH_2-$), 4.21 (2H, m, $-OCH_2-$), 2.91 (2H, m, $-OCH_2-$), 2.80 (3H, s, $-CH_3$), 2.24 (3H, s, $-CH_3$).	MS (ESI) *m/z:* 645.2 $(M + H)^+$, 667.2 $(M + Na)^+$
**28o**	^{1}H-NMR (400 MHz, Acetone-d_6) δ 12.99 (1H, s, 5-OH), 7.94 (1H,	MS (ESI) *m/z:*

	d, *J* = 16.3 Hz, -CH=CHCO-), 7.86 (1H, d, *J* = 8.6 Hz, H-6′), 7.78 (2H, d, *J* = 8.1 Hz, H-2″,6″), 7.75 (1H, s, H-2′), 7.63 (4H, m, H-*o* of diphenylmethane), 7.46 (6H, m, H-*p, m* of diphenylmethane), 7.26 (2H, d, *J* = 7.9 Hz, H-3″,5), 7.20 (1H, d, *J* = 8.8 Hz, H-5′), 7.11 (1H, s, H-3), 6.86 (1H, s, H-8), 6.79 (1H, d, *J* = 15.6 Hz, -CH=CHCO-), 6.68 (1H, s, H-6), 2.30 (3H, s, -CH_3).	657.2 (M + H)+
**28q**	^{1}H-NMR (400MHz, $CDCl_3$) δ 7.70 (1H, d, *J* = 16.0Hz, -CH=CHCO-), 7.56-7.63 (4H, H-*o* of diphenylmethane), 7.53 (2H, d, *J*= 8.4Hz, H-4″, H-6″), 7.45 (1H, dd, *J* = 8.0, 1.6Hz, H-5′), 7.35-7.43 (7H, H-*p, m* of diphenylmethane ,H-2′), 7.11 (2H, d, *J* = 8.8Hz, H-3″, H-5″), 6.99 (1H, d, *J* = 8.0Hz, H-6′), 6.52 (1H, s, H-3), 6.50 (2H, d, *J* = 2.0Hz, H-8), 6.43 (1H, d, J = 16.0Hz, -CH=CHCO-), 6.40 (1H, d, *J* = 2.0Hz, H-6), 4.58 (2H, t, *J* = 4.6Hz, -OCH_2-), 4.31 (2H, t, *J* = 4.4Hz, -OCH_2-), 2.30 (3H, s, CH_3).	MS (ESI) m/z: 683.2 (M + H)+.
**7a**	^{1}H-NMR (400 MHz, Acetone-*d*$_6$) *δ* 13.01(1H, s, 5-OH), 8.88(1H, s, 4′-OH), 8.42(1H, s, 3′-OH), 7.72(1H, d, *J* = 16.0Hz, -CH=CHCO-), 7.70 (2H, s, H-2″,6″), 7.53(1H, s, H-2′), 7.50(1H, s, *J* = 8.0Hz, H-6′), 7.43 (3H, s, H-3″,4″,5″), 7.01 (1H, d, *J* = 8.0Hz, H-5′), 6.76 (1H, s, H-3), 6.62 (1H, s, H-8), 6.60 (1H, d, *J* = 16.0Hz, -CH=CHCO-), 6.39 (1H, s, H-6), 4.60 (2H, t, *J* = 4.4Hz, -OCH_2-), 4.49 (2H, t, *J* = 4.6Hz, -OCH_2-). ^{13}C-NMR (150 MHz, DMSO-*d*$_6$) *δ* 182.23, 166.58, 164.72, 164.39, 161.64, 157.61, 150.25, 146.15, 145.52, 134.33, 131.02, 129.34, 128.88, 121.81, 119.53, 118.07, 116.37, 113.99, 105.26, 103.54, 98.84, 93.59, 67.11, 62.90.	HR-MS (ESI) calcd for $C_{26}H_{20}O_8$: 460.11582, found [M + H]+: 461.12286.
**7b**	^{1}H-NMR (400 MHz, DMSO-*d*$_6$) *δ* 7.87 (1H, d, *J* = 16.2 Hz, -CH=CHCO-), 7.57 (1H, d, *J* = 7.9 Hz, H-6″), 7.41 (1H, d, *J* = 5.8 Hz, H-6′), 7.40(1H, s, H-2′). 7.20 (1H, t, *J* = 7.7 Hz, H-4″), 6.90 (1H, d, *J* = 8.0 Hz, H-3″), 6.81(1H, d, *J* = 8.0 Hz, H-5′), 6.79 (1H, s, H-3), 6.77(1H, t, *J* = 7.7 Hz, H-5″), 6.68(1H, s, H-8), 6.68 (1H, d, *J* = 16.0 Hz, -CH=CHCO-), 6.39 (1H, s, H-6), 4.48 (2H, s, -OCH_2-), 4.40 (2H, s, -OCH_2-). ^{13}C-NMR (150 MHz, DMSO-*d*$_6$) *δ* 182.25, 167.08, 164.73, 164.39, 161.67, 157.62, 157.29, 150.28, 146.19, 141.13, 132.29, 129.45, 121.82, 120.99, 119.84, 119.53, 117.07, 116.58, 116.37, 113.99, 105.27, 103.53, 98.83, 93.57, 67.18, 62.64.	HR-MS (ESI) calcd for $C_{26}H_{20}O_9$: 476.11073, found [M + H]+ :477.11801
**7c**	^{1}H-NMR (400 MHz, DMSO-*d*$_6$) *δ* 12.97 (1H, s, 5-OH), 7.57 (1H, d, *J* = 15.9 Hz, -CH=CHCO-), 7.43 (1H, d, *J* = 10.4 Hz, H-6′), 7.41 (1H, s, H-2′), 7.18 (1H, t, *J* = 7.8 Hz, H-5″), 7.12 (1H, d, *J* = 7.7 Hz, H-6″), 7.02 (1H, s, H-2″), 6.88 (1H, d, *J* = 8.1 Hz, H-5′), 6.82 (1H, d, *J* = 8.3 Hz, H-4″), 6.78 (1H, s, H-3), 6.72 (1H, s, H-8), 6.55 (1H, d, *J* = 16.0 Hz, -CH=CHCO-), 6.41 (1H, s, H-6), 4.49 (2H, s, -OCH_2-), 4.40 (2H, s, -OCH_2-). ^{13}C-NMR (150 MHz, DMSO-*d*$_6$) *δ* 182.24, 166.56, 164.74, 164.40, 161.67, 158.11, 157.61, 150.29, 146.19, 145.72, 135.56, 130.36, 121.81, 119.78, 119.54, 118.20, 117.80, 116.38, 115.19, 113.97, 105.27, 103.52, 98.83, 93.60, 67.11, 62.84.	HR-MS (ESI) calcd for $C_{26}H_{20}O_9$: 476.11073, found[M + H]+: 477.11792.
**7d**	^{1}H-NMR (400 MHz, DMSO-*d*$_6$) *δ* 12.96 (1H, s, 5-OH), 9.96 (1H, s, 4′-OH), 9.59 (1H, s, 4″-OH), 9.36 (1H, s, 3″-OH), 9.10 (1H, s, 3′-OH), 7.49 (d, *J* = 15.9 Hz, -CH=CHCO-), 7.43 (2H, m, H-2′ and H-6′), 7.03 (1H, s, H-2″), 6.99 (1H, d, *J* = 8.2 Hz, H-5″), 6.88 (1H, d, *J* = 8.2 Hz, H-5′), 6.78 (1H, d, *J* = 2.0 Hz, H-8), 6.73 (1H,s, H-3), 6.40 (1H, d, *J* = 2.0 Hz, H-6), 6.30 (1H, d, *J* = 15.9 Hz, -CH=CHCO-), 4.46 (2H, s, -OCH_2-), 4.39 (2H, s, -OCH_2-).	HR-MS (ESI) calcd for $C_{26}H_{20}O_{10}$: 492.10565, found [M + H]+: 493.11334.

	^{13}C-NMR (150 MHz, DMSO-*d*$_6$) δ 182.25, 167.08, 164.73, 164.39, 161.67, 157.62, 157.29, 150.28, 146.19, 141.13, 132.29, 129.45, 121.82, 120.99, 119.84, 119.53, 117.07, 116.58, 116.37, 113.99, 105.27, 103.53, 98.83, 93.57, 67.18, 62.64.	
**7e**	^{1}H-NMR (400 MHz, DMSO-*d*$_6$) δ 12.96 (1H, s, 5-OH), 9.97 (1H, s, 4′-OH), 9.35 (1H, s, 3′-OH), 7.80 (1H, m, H-3″,5″), 7.67 (d, *J* = 16.0 Hz, -<u>CH</u>=CHCO-), 7.42 (1H, d, *J* = 9.5 Hz, H-6′), 7.41 (1H, s, H-2′), 7.22 (2H, t, *J* = 8.7 Hz, H-2″,6″), 6.88 (1H, d, *J* = 8.2 Hz, H-5′), 6.77 (1H, s, H-3), 6.71 (1H, s, H-8), 6.66 (1H, d, *J* = 16.0 Hz, -CH=<u>CH</u>CO-), 6.40 (1H, s, H-6), 4.50 (2H, s, -OCH$_2$-), 4.40 (2H, s, -OCH$_2$-). ^{13}C-NMR (101 MHz, DMSO-*d*$_6$) δ 182.47, 172.66, 166.11, 165.01, 164.56, 161.91, 157.86, 150.59, 146.47, 121.99 (2), 119.72 (2), 116.72, 114.33, 105.54, 103.74, 99.06, 93.78, 67.31, 63.51.	HR-MS (ESI) calcd for $C_{26}H_{19}FO_8$: 478.10640, found [M + H]$^+$: 479.11325.
**7f**	^{1}H-NMR (400 MHz, DMSO-*d*$_6$) δ 12.96 (1H, s, 5-OH), 9.97 (1H, s, 4′-OH), 9.35 (1H, s, 3′-OH), 7.75 (2H, d, *J* = 7.5 Hz, H-2″,6″), 7.66 (1H, d, *J* = 16.0 Hz, -<u>CH$_2$</u>=CH$_2$CO-), 7.45(2H, d, *J* = 7.7 Hz, H-3″,5″),7.44(1H, d, *J* = 8.0Hz, H-6′), 7.41(1H, s, H-2′), 6.88 (1H, d, *J* = 8.3 Hz, H-5′), 6.78 (1H, s, H-3), 6.72 (1H, s, H-8), 6.72(1H, d, *J* = 16.0 Hz, -CH=<u>CH</u>CO-), 6.40 (1H, s, H-6), 4.50 (2H, s, -OCH$_2$-), 4.40 (2H, s, -OCH$_2$-). ^{13}C-NMR (150 MHz, DMSO-*d*$_6$) δ 182.25, 166.46, 164.73, 164.39, 161.61, 157.60, 150.29, 146.17, 144.09, 135.53, 133.24, 130.57, 129.38, 121.76, 119.55, 118.86, 116.38, 113.88, 105.24, 103.46, 98.84, 93.60, 67.09, 62.98.	HR-MS (ESI) calcd for $C_{26}H_{19}ClO_8$: 494.07685, found [M + H]$^+$: 495.08334.
**7g**	^{1}H-NMR (400 MHz, DMSO-*d*$_6$) δ 12.98 (1H,s,5-OH), 10.00 (1H, s, 4′-OH), 9.38 (1H, s, 3′-OH), 8.22 (2H, d, *J* = 7.9 Hz, H-3″, 5″), 8.02 (2H, d, *J* = 7.9 Hz, H-2″, 6″), 7.80 (1H, d, *J* = 15.8 Hz, -<u>CH$_2$</u>=CH$_2$CO-), 7.42 (1H, d, *J* = 8.2 Hz, H-6′), 7.41(1H, s, H-2′), 6.94 (1H, d, *J* = 16.2 Hz, -CH=<u>CH</u>CO-), 6.90 (1H, d, *J* = 8.5 Hz, H-5′), 6.80 (1H, s, H-3), 6.74 (1H, s, H-8), 6.43 (1H, s, H-6), 4.56 (2H, s, -OCH2-), 4.43 (2H, s, -OCH2-). ^{13}C-NMR (150 MHz, DMSO-*d*$_6$) δ 182.25, 166.07, 164.73, 164.36, 161.66, 157.60, 150.29, 148.55, 146.19, 142.90, 141.08, 140.75, 130.66, 129.98, 124.34, 123.44, 123.12, 122.41, 121.79, 119.53, 116.36, 113.97, 105.28, 103.51, 98.86, 93.58, 93.40, 67.07, 63.22.	HR-MS (ESI) calcd for $C_{26}H_{19}NO_{10}$: 505.10090, found [M + H]$^+$: 506.10837.
**7h**	^{1}H-NMR (400 MHz, DMSO-*d*$_6$) δ 12.96 (1H, s, 5-OH), 9.97 (1H, s, 4′-OH), 9.36 (1H, s, 3′-OH), 7.53 (1H, d, *J* = 16.0 Hz, -<u>CH</u>=CHCO-), 7.50 (2H, d, *J* = 8.9 Hz, H-3″, 5″), 7.43 (1H, d, *J* = 10.6 Hz, H-6′), 7.41 (1H, s, H-2′), 6.88 (1H, d, *J* = 8.1 Hz, H-5′), 6.78 (1H, d, *J* = 1.9 Hz, H-8), 6.72 (1H, s, H-3), 6.65 (2H, d, *J* = 8.8 Hz, H-2″, 6″), 6.40 (1H, d, *J* = 2.0 Hz, H-6), 6.33 (1H, d, *J* = 15.8 Hz, -CH=<u>CH</u>CO-), 4.44 (2H, s, -OCH$_2$-), 4.39 (2H, s, -OCH$_2$-). ^{13}C-NMR (150 MHz, DMSO-*d*$_6$) δ 182.24, 167.20, 164.73, 164.47, 161.66, 157.61, 152.25, 150.29, 146.20, 133.08, 130.48, 130.02, 129.11, 129.01, 126.20, 121.83, 121.63, 119.54, 116.38, 114.00, 112.13, 111.56, 105.26, 103.53, 98.87, 93.58, 67.26, 62.43..	HR-MS (ESI) [M + H]$^+$ calcd for $C_{28}H_{25}NO_8$: 503.15802, found: 504.16522
**7i**	^{1}H-NMR (400 MHz, DMSO-*d*$_6$) δ 7.64 (2H, d, *J* = 8.4 Hz, H-3″, 5″), 7.60 (1H, d, *J* = 16.0 Hz, -<u>CH</u>=CHCO-), 7.43 (1H, s, H-2′), 7.42 (1H, d, *J* = 8.4 Hz, H-6′), 6.92(2H, d, *J* = 8.4 Hz, H-2″, 6″) 6.91 (1H, d, *J* = 8.1 Hz, H-5′), 6.76 (1H, d, *J* = 1.7 Hz, H-8), 6.69 (1H, s, H-3), 6.51 (1H, d, *J* = 15.9 Hz, -CH=<u>CH</u>CO-), 6.38 (1H, d, *J* = 1.7 Hz, H-6), 4.47 (2H, s, -OCH$_2$-), 4.39 (2H, s, -OCH$_2$-), 3.76	HR-MS (ESI) calcd for $C_{27}H_{22}O_9$: 490.12638, found[M + H]$^+$: 491.13263.

	(3H, s, -CH_3). ^{13}C-NMR (101 MHz, DMSO-d_6) δ 182.46, 167.07, 164.99, 164.64, 161.89, 157.84, 150.57, 146.46, 145.53, 130.91, 127.20, 122.03, 119.72, 116.71, 115.55, 115.03, 114.32, 105.51, 103.73, 93.79, 67.41, 62.95, 56.00.	
**7j**	^{1}H-NMR (400 MHz, DMSO-d_6) δ 12.93 (1H, s, 5-OH), 9.92 (1H, s, 4′-OH), 9.33 (1H, s, 3′-OH), 7.63 (1H, d, *J* = 16.0 Hz, -CH_2=CH_2CO-), 7.62 (2H, d, *J* = 8.4 Hz, H-2″, 6″), 7.42 (t, *J* = 6.6 Hz, 1H), 7.41(1H, d, *J* = 8.4 Hz, H-6′), 7.40(1H, s, H-2′) , 7.39(2H, d, *J*= 7.4Hz, H-3″, 5″)， 6.88 (1H, d, *J* = 8.0 Hz, H-5′), 6.79 (1H, s, H-3), 6.71 (1H, s, H-8), 6.62 (1H, d, *J* = 16.3 Hz, -CH=CHCO-), 6.41 (1H, s, H-6), 4.49 (2H, s, -OCH_2-), 4.40 (2H, s, -OCH_2-), 1.26 (3H, s, -CH_3). ^{13}C-NMR (150 MHz, DMSO-d_6) δ 182.24, 166.68, 164.73, 164.41, 161.66, 157.61, 153.95, 150.29, 146.19, 145.38, 131.62, 128.72, 126.16, 121.80, 119.54, 117.13, 116.36, 113.98, 105.27, 103.52, 98.87, 93.58, 67.13, 62.87, 40.47, 31.30 (3).	HR-MS (ESI) calcd for $C_{30}H_{28}O_8$: 516.17842, found[M + H]$^+$: 517.17853.
**7k**	^{1}H-NMR (400 MHz, DMSO-d_6) δ 9.16 (1H, s, H-2″), 8.80 (1H, d, *J* = 5.1 Hz, H-4″), 8.73 (1H, d, *J* = 6.6 Hz, H-6″), 7.89 (1H, d, *J* = 7.4 Hz, H-5′), 7.80 (1H, d, *J* = 16.1 Hz, -CH_2=CH_2CO-), 7.43 (1H, s, H-2′), 7.40(1H, d, *J* = 8.0 Hz, H-6′) , 7.04 (1H, d, *J* = 16.2 Hz, -CH=CHCO-)), 6.91 (1H, d, *J* = 8.0 Hz, H-5′), 6.76 (1H, d, *J* = 1.6 Hz, H-8), 6.70 (s, 1H), 6.38 (1H, d , *J* = 1.6 Hz, H-6), 4.54 (2H, s, -OCH_2-), 4.40 (2H, s, -OCH_2-). ^{13}C-NMR (150 MHz, DMSO-d_6) δ 182.22, 172.40, 165.90, 164.75, 164.31, 161.65, 157.60, 150.32, 146.20, 146.00, 145.27, 140.82, 140.03, 132.51, 126.39, 122.68, 121.76, 119.47, 116.45, 114.07, 105.29, 103.50, 98.81, 93.53, 67.07, 63.24.	HR-MS (ESI) calcd for C25H19NO8: 461.11107, found[M + H]$^+$: 462.11722.
**7l**	^{1}H NMR (400 MHz, DMSO-d_6) δ 8.09 (1H, s, H-2″), 7.71 (1H, s, H-5″), 7.59 (1H, d, *J* = 15.8 Hz, -CH_2=CH_2CO-), 7.42 (2H, m, H-2′, 6′), 6.97 (1H, s, H-3″), 6.87 (1H, d, *J* = 8.2 Hz, H-5′), 6.75(1H, s, H-3) , 6.73(1H, s , H-8), 6.42 (1H, d, *J* = 16.2 Hz, -CH=CHCO-), 6.41(1H, s, H-6). ^{13}C-NMR (150 MHz, DMSO-d_6) δ 182.24, 166.62, 164.75, 164.39, 161.66, 157.61, 150.34, 146.45, 146.20, 145.44, 136.09, 122.79, 121.78, 119.54, 117.49, 116.38, 113.96, 108.35, 105.27, 103.50, 98.80, 93.55, 67.15, 62.68.	HR-MS (ESI) [M + H]$^+$ calcd for $C_{24}H_{18}O_9$: 450.09508, found[M + H]$^+$: 451.10138.
**7m**	^{1}H-NMR (400 MHz, DMSO-d_6) δ 7.42 (1H, d, *J* = 8.4 Hz, H-6′), 7.41 (1H, s, H-2′), 6.87 (1H, d, *J* = 8.1 Hz, H-5′), 6.81 (1H, dd, *J* = 15.8, 6.8 Hz, -CH_2=CH_2CO-), 6.75 (1H, s, H-3), 6.70 (1H, s, H-8), 6.37 (1H, d, *J* = 1.6 Hz, H-6), 5.79 (d, *J* = 15.8 Hz, -CH=CHCO-), 4.39 (2H, m, -OCH_2-), 4.36 (2H, m, -OCH_2-), 2.52 – 2.45 (1H, m, -CH), 1.72 – 1.64 (4H, m, -CH_2-), 1.32 – 1.02 (6H, m, -CH_2-). ^{13}C-NMR (150 MHz, DMSO-d_6) δ 182.20, 166.25, 164.75, 164.43, 161.64, 157.57, 155.32, 150.47, 146.26, 121.68, 119.51, 118.71, 116.37, 113.91, 105.25, 103.44, 98.89, 93.56, 67.01, 62.71, 40.38, 39.96, 39.68, 39.54, 25.80, 25.57.	HR-MS (ESI) calcd for $C_{26}H_{26}O_8$: 466.16277, found [M + H]$^+$: 467.16916.
**7n**	^{1}H-NMR (400 MHz, DMSO-d_6) δ 7.47 – 7.43 (2H, m, H-2′, 6′), 6.94 (1H,d, J=8.1Hz, H-5′), 6.78 (1H, s, H-4″),6.72 (1H, s, H-3)6.40 (1H, s, H-8), 5.75 (1H, s, H-6), 4.39 (1H, m, -CH_2-), 4.33 (1H, m, -CH_2-), 4.20 – 4.09 (1H, m, -CH_2-), 2.94 – 2.83 (1H, m, -CH_2-), 2.22 (3H, s, -CH_3), 2.06 (3H, s, -CH_3). ^{13}C-NMR (150 MHz, DMSO-d_6) δ 182.22, 171.28, 164.76, 164.32, 161.65, 157.58, 150.47, 146.36, 146.25, 139.12, 121.69, 119.54, 116.38, 113.93, 105.25, 104.85, 103.47, 98.77, 93.52, 66.98, 62.79, 43.55, 40.38,	HR-MS (ESI) calcd for $C_{25}H_{24}N_2O_8$: 480.15327, found[M + H]$^+$: 481.16052.

	39.69, 39.55.	
**7o**	^{1}H-NMR (500 MHz, DMSO-d_6) δ 7.82 (2H, d, J = 8.4 Hz, H-2″, 6″), 7.45 (1H, d, J = 8.2 Hz, H-6′), 7.45 (1H, s, H-2′), 6.91 (1H, d, J = 8.3 Hz, H-5′), 6.84 (2H, d, J = 8.3 Hz, H-3″, 5″), 6.81 (1H, s, H-3), 6.74 (1H, s, H-8), 6.43 (1H, s, H-6), 4.57 (2H, m, -OCH_2-), 4.47 (2H, m, -OCH_2-). ^{13}C-NMR (150 MHz, DMSO-d_6) δ 182.23, 165.87, 165.74,164.50, 164.38, 162.5, 157.60, 156.34, 152.34, 146.20, 131.97, 121.81, 120.40, 119.51, 116.40, 115.78, 114.01, 108.71, 105.25, 105.21, 103.50, 102.81, 98.89, 93.67, 67.19, 62.97.	HR-MS (ESI) calcd for $C_{24}H_{18}O_9$: 450.09508, found [M + H]$^+$: 451.10220.
**7p**	^{1}H-NMR (400 MHz, DMSO-d_6) δ 7.43 (1H, d, J = 8.5 Hz, H-6′), 7.41 (1H, s, H-2′), 6.98 (1H, s, H-3), 6.96 (1H, s, H-8), 6.88 (1H, d, J = 8.1 Hz, H-5′), 6.73 (2H, d, J = 8.9 Hz, H-3″, 5″), 6.60 (2H, d, J = 8.1 Hz, H-2″, 6″), 6.37 (1H, s, H-6), 4.33 (2H, s, -OCH_2-), 4.30 (2H, s, -OCH_2-), 2.72 (2H, t, J = 7.4 Hz, -CH_2-), 2.57 (2H, t, J = 7.3 Hz, -CH_2-).^{13}C-NMR (150 MHz, DMSO-d_6) δ 182.24, 172.67, 164.74, 164.35, 161.65, 157.60, 156.01, 150.39, 146.22(2), 130.84, 129.53, 121.73(2), 119.55, 116.37, 115.47, 113.95, 105.25, 103.49, 98.79, 93.52, 67.05, 62.57, 40.48, 40.36.	HR-MS (ESI) calcd for $C_{26}H_{22}O_9$: 478.12638, found [M + H]$^+$:479.13336.
**7q**	^{1}H-NMR (400 MHz, DMSO-d_6) δ 12.96 (1H, s, 5-OH), 9.97 (1H, s, 4'-OH), 9.36 (1H, s, 3'-OH), 7.76 (2H, d, J = 8.3 Hz, H-2'', 6''), 7.67 (1H, d, J = 16.0 Hz, -CH=CHCO-), 7.41 (1H, d, J = 8.1Hz, H-6'), 7.41 (1H, s, H-2′), 7.15 (2H, d, J = 8.1 Hz, H-3'', 5''), 6.88 (1H, J = 8.0Hz, H-5'), 6.77 (1H, s, H-3), 6.71 (1H, s, H-8), 6.67 (1H, d, J = 16.1 Hz, -CH=CHCO-), 6.40 (1H, s, H-6), 4.50 (2H, s, -OCH_2-), 4.40 (2H, s, -OCH_2-), 2.25 (3H, s, -CH_3). ^{13}C-NMR (101 MHz, DMSO-d_6) δ 182.49, 169.66, 166.80, 164.98, 164.67, 161.93, 157.86, 152.76, 150.54, 146.45, 144.78, 132.28, 130.40, 123.05, 122.08 (2), 119.69, 118.38, 116.63, 114.25 (2), 109.99, 103.78, 93.81, 67.36, 63.14, 21.55.	HR-MS (ESI) calcd for $C_{28}H_{22}O_{10}$: 518.12130, found [M + H]$^+$: 519.12750.

The synthesis of other compounds

1.1. Synthesis of ***Series*** *I compounds*

1.1.1. Synthesis of compound 4a–4h

1-(2-hydroxy-4,6-bis(methoxymethoxy)phenyl)ethanone, 9

The starting material 2′4′6′-trihydroxyacetophenone trihydrate (930 mg, 5 mmol) was dissolved in DCM (20 mL), and MOMCl (0.94 mL, 12.5 mmol) was added at -10°C. After 10 mins stirring, DIEA (2.2mL, 12.5mmol) was added slowly and the suspension heated to 0°C. Saturated sodium bicarbonate (20 mL) was added to quench reaction after 1h. The dichloromethane layer was separated, then the aqueous phase was extracted with dichloromethane (20 mL). The organic phase was merged, then washed with water and brine, dried over Na_2SO_4, filtered and concentrated *in vacuo*. The residue was purified by column chromatography (petroleum ether/acetone = 20:1) to give **9** as white solids (1.1 g, 85.9%).^{1}H NMR (400 MHz, $CDCl_3$) δ 6.25 (1H, d, J = 2.4 Hz, H-2), 6.22 (1H, d, J = 2.4 Hz, H-5), 5.24 (2H, s, -CH_2-), 5.15 (2H, s, -CH_2-), 3.50 (3H, s,-OCH_3), 3.45 (3H, s, -OCH_3), 2.64(3H, s, -$COCH_3$). MS (ESI) *m/z:* 257.1(M + H)$^+$. The spectroscopic data of **9** were consistent with the reference. [S1]

3-((2-(trimethylsilyl)ethoxy)methoxy)benzaldehyde, 10a

Compound **10a** (167 mg, 66.3%) was synthesized from **3-hydroxybenzaldehyde** (122 mg, 1 mmol) and SEMCl (213 μL, 1.2 mmol) according to the procedure used to prepare **9** (petroleum ether/ethyl acetate = 40:1), obtained as colorless oil. ^{1}H-NMR (400 MHz, $CDCl_3$) *δ* 9.97 (1H, s, -CHO), 7.54 – 7.52 (1H, m, H-2), 7.50 (1H, dd, *J* = 8.4, 0.9 Hz, H-6), 7.44 (1H, t, *J* = 7.8 Hz, H-5), 7.31 – 7.26 (1H, m, H-4), 5.26 (1H, s, $-OCH_2O-$), 3.75 (2H, t, *J* = 8.6, $-O\underline{CH_2}CH_2Si-$), 0.95 (1H, t, J=8.1 Hz, $-OCH_2\underline{CH_2}Si-$), -0.02 (9H, s,$-CH_3$). MS (ESI) *m*/*z*: 274.3(M + Na)$^+$. The spectroscopic data of **10a** were consistent with the reference. [S2]

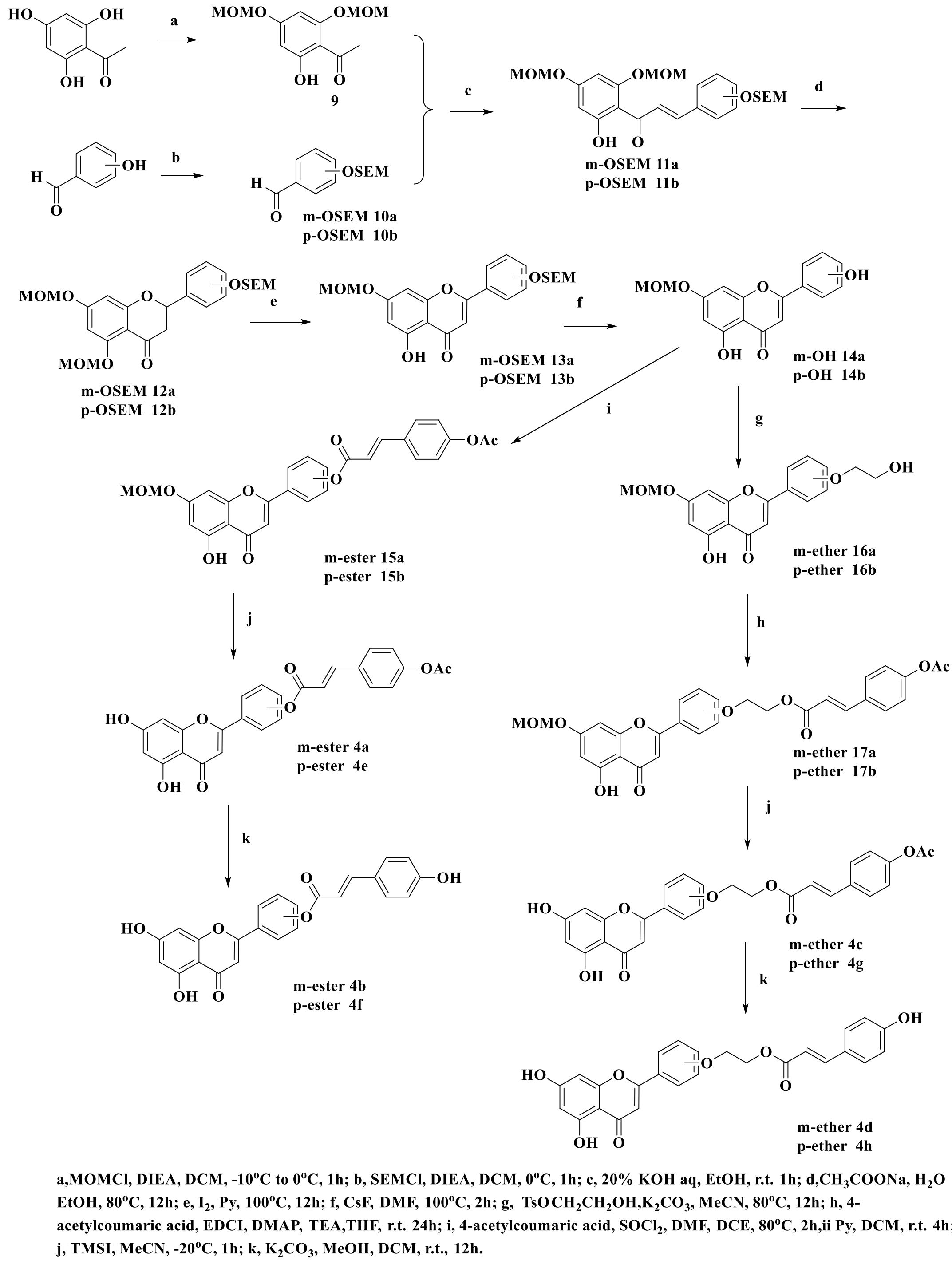

a,MOMCl, DIEA, DCM, -10°C to 0°C, 1h; b, SEMCl, DIEA, DCM, 0°C, 1h; c, 20% KOH aq, EtOH, r.t. 1h; d,CH_3COONa, H_2O EtOH, 80°C, 12h; e, I_2, Py, 100°C, 12h; f, CsF, DMF, 100°C, 2h; g, $TsOCH_2CH_2OH$,K_2CO_3, MeCN, 80°C, 12h; h, 4-acetylcoumaric acid, EDCI, DMAP, TEA,THF, r.t. 24h; i, 4-acetylcoumaric acid, $SOCl_2$, DMF, DCE, 80°C, 2h,ii Py, DCM, r.t. 4h; j, TMSI, MeCN, -20°C, 1h; k, K_2CO_3, MeOH, DCM, r.t., 12h.

Scheme S1. Synthesis of compound 4a–4h.

4-((2-(trimethylsilyl)ethoxy)methoxy)benzaldehyde, 10b

Compound **10b** (116 mg, 45.8%) was synthesized from **4-hydroxybenzaldehyde** (122 mg, 1 mmol) and SEMCl (213 μL, 1.2 mmol） according to the procedure used to prepare **9** (petroleum ether/ethyl acetate = 40:1), obtained as colorless oil. ^{1}H-NMR (400 MHz,$CDCl_3$) *δ* 9.88 (1H, s, -CHO), 7.81 (2H, d, J=8.0Hz, H-3,5), 7.13 (2H, d, J=8.0Hz, H-2,6), 5.28 (1H, s, -OCH_2O-), 3.75 (2H, t, *J* = 8.6, -O$CH_2$$CH_2$Si-), 0.95 (1H, t, J=8.1 Hz, -$OCH_2$$CH_2$Si-), -0.02 (9H, s, -$CH_3$). MS (ESI) *m/z:* 269.2(M + H)$^+$ The spectroscopic data of **10b** were consistent with the reference. [S2]

(*E*)-1-(2-hydroxy-4,6-bis(methoxymethoxy)phenyl)-3-(3-((2-(trimethylsilyl)ethoxy)methoxy)phenyl)prop-2-en-1-one, 11a

To a mixture of acetophenone **9** (57 mg, 0.22 mmol) and benzaldehyde 1**0a** (56 mg, 0.22 mmol) in ethanol (1 mL) was added dropwise 20% w/v solution of potassium hydroxide (1 mL) at 0°C. The mixture was stirred at room temperature for 12 h. The residue was acidified with 1M HCl solution to no more turbidity (the pH was nearly 13), and extracted with CH_2Cl_2 (5mL*3). The organic layer was washed with brine, dried over Na_2SO_4, filtered and concentrated *in vacuo*. The crude product was purified by column chromatography (PE/acetone = 15:1) to give 1**1a** as yellow solids (74 mg, 68.6%). ^{1}H-NMR (400 MHz, $CDCl_3$) *δ* 7.82 (1H, d, J =16.0Hz, -CH=CHCO-), 7.78 (1H, d, J =16.0Hz , -CH=CHCO-), 7.54 – 7.52 (1H, m, H-2), 7.50 (1H, dd, *J* = 8.4, 0.9 Hz, H-6), 7.44 (1H, t, *J* = 7.8 Hz, H-5), 6.31 (1H, d, *J* = 1.9 Hz, H-8), 6.24 (1H, d, *J* = 1.6 Hz, H-6), 5.28(2H, s, -OCH_2O-), 5.25 (2H,s, -OCH_2O-), 5.18 (2H,s, -OCH_2O-), 3.75 (2H, t, *J* = 8.2 Hz, -O$CH_2$$CH_2$Si-),, 3.53 (3H, s, -$OCH_3$), 3.47 (3H, s, -$OCH_3$), 0.96 (2H, t, *J* = 8.2 Hz, -$OCH_2$$CH_2$Si-), -0.01 (9H, s, -Si($CH_3$)$_3$). MS (ESI) *m/z:* 507.2(M + H)$^+$

(*E*)-1-(2-hydroxy-4,6-bis(methoxymethoxy)phenyl)-3-(4-((2-(trimethylsilyl)ethoxy)methoxy)phenyl)prop-2-en-1-one, 11b

Compound **11b** (1.3 g, 55.7%) was synthesized from **10b** (1.2 g, 4.8 mmol) according to the procedure used to prepare **11a**, obtained as yellow solids. ^{1}H-NMR (400 MHz, $CDCl_3$) *δ* 7.82 (1H, d, J =16.0Hz, -CH=CHCO-), 7.78 (1H, d, J =16.0Hz , -CH=CHCO-), 7.54 (2H, d, *J* = 8.5 Hz, H-3′, 5′), 7.06 (2H, d, *J* = 8.4 Hz, H-2′,6′), 6.31 (1H, d, *J* = 1.9 Hz, H-8), 6.24 (1H, d, *J* = 1.6 Hz, H-6), 5.28(2H,s, -OCH_2O-), 5.25 (2H,s, -OCH_2O-), 5.18 (2H,s, -OCH_2O-), 3.75 (2H, t, *J* = 8.2 Hz, -O$CH_2$$CH_2$Si-),, 3.53 (3H, s, -$OCH_3$), 3.47 (3H, s, -$OCH_3$), 0.96(2H, t, *J* = 8.2 Hz, -$OCH_2$$CH_2$Si-), -0.01 (9H, s, -Si($CH_3$)$_3$). MS (ESI) *m/z:* 507.2(M + H)$^+$

5,7-bis(methoxymethoxy)-2-(3-((2-(trimethylsilyl)ethoxy)methoxy)phenyl)chroman-4-one, 12a

A solution of chalcone **11a** (74 mg, 0.15 mmol) and sodium acetate (123 mg, 1.5 mmol) in a mixture of ethanol/H_2O (3:1, v/v, 4 mL) was stirred under refluxing for 8 h, and then water (6 mL) was added. The residue was extracted with ethyl acetate, washed with brine, dried over Na_2SO_4, filtered and concentrated *in vacuo*. The crude product was purified by column chromatography (PE/acetone = 10:1) to give 1**2a** as yellow solid (61mg, 82.4%). ^{1}H-NMR (500 MHz, $CDCl_3$) *δ* 7.37 (1H, t, *J* = 7.9 Hz, H-5′), 7.18 (1H, s, H-2′), 7.13 – 7.07 (2H, m, H-4′,6′), 6.48 (1H, s, H-8), 6.45 (1H, s, H-6), 5.42 (1H, d, *J* = 13.2 Hz, H-2), 5.32 (2H, s, -OCH_2O-), 5.29 (2H, s, -OCH_2O-), 5.22 (2H,s, -OCH_2O-), 3.81 (2H, t, *J* = 8.2 Hz, -O$CH_2$$CH_2$Si-), 3.58 (3H, s, -$OCH_3$), 3.52 (3H, s, -$OCH_3$), 3.04 (1H, t, *J* = 14.9 Hz, H-3b), 2.83 (1H, d, *J* = 16.5 Hz, H-3a), 1.00 (2H, t, *J* = 8.2 Hz, -$OCH_2$$CH_2$Si-), 0.05 (9H, s, -Si($CH_3$)$_3$). MS (ESI) *m/z:* 446.2(M + H)$^+$

5,7-bis(methoxymethoxy)-2-(4-((2-(trimethylsilyl)ethoxy)methoxy)phenyl)chroman-4-one, 12b

Compound **12b** (343 mg, 34.3%) was synthesized from **12b** (1 g, 2 mmol) according to the procedure used to prepare **12a**, obtained as yellow solids, and **11b** (262 mg, 26.2%) was recycled. ^{1}H-NMR (400 MHz, $CDCl_3$) *δ* 7.36 (2H, d, *J* = 8.7 Hz, H-3′,5′), 7.07 (2H, d, *J* = 8.5 Hz, H-2′,6′), 6.42 (1H, d, *J* = 2.3 Hz, H-8), 6.37 (1H, d, *J* = 2.3, H-6), 5.37 – 5.32 (1H, m, H-2) , 5.22 (2H, s, -OCH_2O-), 5.15 (2H, s, -OCH_2O-), 3.75 (2H, t, *J* = 8.2 Hz, -O$CH_2$$CH_2$Si-), 3.52 (3H, s, -$OCH_3$), 3.01 (1H, dd, *J* = 16.5, 13.3 Hz, H-3b), 2.75 (1H, dd, *J* = 16.5, 2.8 Hz, H-3a), 0.95 (2H, t, *J* = 8.2 Hz, -$OCH_2$$CH_2$Si-), -0.01 (9H, s, -Si($CH_3$)$_3$). MS (ESI) *m/z:* 446.2(M + H)$^+$

5-hydroxy-7-(methoxymethoxy)-2-(3-((2-(trimethylsilyl)ethoxy)methoxy)phenyl)-4*H*-chromen-4-one, 13a

A solution of flavanone 1**2a** (35 mg, 0.07 mmol) and iodine (18 mg, 0.07 mmol) in dry pyridine (1 mL) was heated to 100°C for 8 h. The resulting mixture was diluted with water(7mL) and extracted with ethyl acetate (10 mL*2). The organic phase was washed by saturated sodium thiosulfate, $CuSO_4$ solution and brine, successively. The organic layer was dried over Na_2SO_4, filtered and concentrated *in vacuo*. The crude product was purified by column chromatography (petroleum ether/acetone = 12:1) to give **13a** as yellow solid (17 mg, 54.7%). ^{1}H-NMR (400 MHz, $CDCl_3$) *δ* 12.66 (1H, s, 5-OH), 7.54 (1H, s, H-2′), 7.52 (1H, m, H-6′), 7.41 (1H, t, *J* = 8.0 Hz, H-5′), 7.22 (1H, dd, *J* = 8.1, 1.6 Hz, H-4′), 6.67 (1H, d, *J* = 2.1 Hz, H-8), 6.66 (1H, s, H-3), 6.48 (1H, d, *J* = 2.2 Hz, H-6), 5.28 (2H,s, $-OCH_2O-$), 5.24(2H,s, $-OCH_2O-$), 3.78 (2H, t, *J* = 8.2 Hz, $-O\underline{CH_2}CH_2O-$),, 3.50 (1H, s, $-CH_3$), 0.97 (2H, t, *J* = 8.2 Hz, $-OCH_2\underline{CH_2}-$) -0.02 (9H, s, -CH3). MS (ESI) *m/z:* 461.2(M + H)$^+$

5-hydroxy-7-(methoxymethoxy)-2-(4-((2-(trimethylsilyl)ethoxy)methoxy)phenyl)-4*H*-chromen-4-one, 13b

Compound **13b** (140 mg, 87.6%) was synthesized from **12b** (175 mg, 0.36 mmol) according to the procedure used to prepare **13a**, obtained as yellow solids. ^{1}H-NMR (400 MHz, $CDCl_3$) *δ* 12.66 (1H, s, 5-OH), 7.54 (1H, s, H-2′), 7.52 (1H, m, H-6′), 7.41 (1H, t, *J* = 8.0 Hz, H-5′), 7.22 (1H, dd, *J* = 8.1, 1.6 Hz, H-4′), 6.67 (1H, d, *J* = 2.1 Hz, H-8), 6.66 (1H, s, H-3), 6.48 (1H, d, *J* = 2.2 Hz, H-6), 5.28 (2H,s, $-OCH_2O-$), 5.24(2H,s, $-OCH_2O-$) 3.78 (2H, t, *J* = 8.2 Hz, $-O\underline{CH_2}CH_2O-$), 3.50 (1H, s, $-CH_3$), 0.97 (2H, t, *J* = 8.2 Hz, $-OCH_2\underline{CH_2}-$), -0.02 (9H, s, -CH3). MS (ESI) *m/z:* 461.2(M + H)$^+$

5-hydroxy-2-(3-hydroxyphenyl)-7-(methoxymethoxy)-4*H*-chromen-4-one, 14a

A solution of compound 1**3a** (178 mg, 0.4 mmol) and CsF (304 mg, 2 mmol) in dry DMF (2 mL) was heated to 100°C for 2 h. The resulting mixture was diluted with water (5mL) and extracted with ethyl acetate (10mL*3). The organic layer was dried over Na_2SO_4, filtered and concentrated *in vacuo*. The crude product was purified by column chromatography (petroleum ether/acetone = 5:1) to give **14a** as yellow solid (94 mg, 74.8%). ^{1}H-NMR (500 MHz, DMSO-d_6) *δ* 12.95 (1H, s, 5-OH), 10.39 (1H, s, 4′-OH), 7.54 (1H, s, H-2′), 7.52 (1H, m, H-6′), 7.41 (1H, t, *J* = 8.0 Hz, H-5′), 7.22 (1H, dd, *J* = 8.1, 1.6 Hz, H-4′)6.86 (1H, s, H-3), 6.82 (1H, d, *J* = 1.5 Hz, H-8), 6.44 (1H, d, *J* = 1.5 Hz, H-6), 5.32 (2H, s, $-OCH_2O-$), 3.42 (3H, s, $-CH_3$). MS (ESI) *m/z:* 331.2(M + H)$^+$. The spectroscopic data of **14a** were consistent with the reference. [S3]

5-hydroxy-2-(4-hydroxyphenyl)-7-(methoxymethoxy)-4*H*-chromen-4-one, 14b

Compound **14b** (74 mg, 78.6%) was synthesized from **13b** (134 mg, 0.3 mmol) according to the procedure used to prepare **14a**, obtained as yellow solids. ^{1}H-NMR (500 MHz, DMSO-d_6) *δ* 12.95 (1H, s, 5-OH), 10.39 (1H, s, H-4′), 7.97 (2H, d, *J* = 8.7 Hz, H-3′,5′), 6.94 (2H, d, *J* = 8.5 Hz, H-2′,6′), 6.86 (1H, s, H-3), 6.82 (1H, d, *J* = 1.5 Hz, H-8), 6.44 (1H, d, *J* = 1.5 Hz, H-6), 5.32 (2H, s, $-OCH_2O-$), 3.42 (3H, s, $-CH_3$). MS (ESI) *m/z:* 331.2(M + H)$^+$

(*E*)-3-(5-hydroxy-7-(methoxymethoxy)-4-oxo-4*H*-chromen-2-yl)phenyl-3-(4-acetoxyphenyl)acrylate, 15a

To a solution of 4-acetyl cinnamic acid (0.4 mg, 0.21 mmol) in dichloroethane (5mL) was added $SOCl_2$ (0.8 mL, 10 mmol) and DMF (1d). The solution was stirred at 80°C for 1h and the organic solvent was removed under vacuum and the residue dissolved in dry CH_2Cl_2 (4 mL). Pyridine (31 μL, 0.38 mmol) was added at 0°C, then **14a** (30 mg, 0.96 mmol) was added dropwise. The resulting mixture was transferred to r.t. and stirred for 4 h. The organic solvent was washed with 1M hydrochloric acid and brine, dried over Na_2SO_4, filtered and concentrated *in vacuo*. The crude product was purified by column chromatography (petroleum ether/DCM/acetone = 10:10:1) to give **15a** as yellow solid (20 mg, 41.5%). ^{1}H-NMR (500 MHz, Acetone-d_6) *δ* 12.81 (1H, s, 5-OH), 8.04 (1H, d, *J* = 7.9 Hz, H-6′), 7.98 (1H, t, *J* = 1.9 Hz, H-2′), 7.96 (1H, d, *J* = 16.2 Hz, $-\underline{CH}=CHCO-$), 7.88 (2H, d, *J* = 8.6 Hz, H-2″,6″), 7.70 (1H, t, *J* = 8.0 Hz, H-5′), 7.50 (1H, dd, *J* = 7.7, 1.9 Hz, H-4′), 7.28 (1H, d, *J* = 8.8 Hz,

H-3″,5″), 6.93 (1H, s, H-3), 6.85 (1H, d, *J* = 2.2 Hz, H-8), 6.83 (1H, d, *J* = 16.0 Hz, -CH=CHCO-), 6.46 (1H, d, *J* = 2.1 Hz, H-6), 5.36 (2H, s, -OCH_2O-).,3.55 (3H, s, -OCH_3), 2.36 (3H, s, -$COCH_3$). MS (ESI) *m/z:* 519.2(M + H)$^+$

(*E*)-4-(5-hydroxy-7-(methoxymethoxy)-4-oxo-4*H*-chromen-2-yl)phenyl-3-(4-acetoxyphenyl)acrylate, 15b

Compound **15b** (13 mg, 24.0%) was synthesized from **14b** (26 mg, 0.083 mmol) according to the procedure used to prepare **15a**, obtained as light-yellow solids. ^{1}H-NMR (500 MHz, $CDCl_3$) *δ* 12.68 (1H, s, 5-OH), 7.97 (2H, d, *J* = 8.8 Hz, H-2′,6′), 7.91 (1H, d, *J* = 15.9 Hz, -CH=CHCO-), 7.65 (2H, d, *J* = 8.6 Hz, H-2″,6″), 7.39 (1H, d, *J* = 8.8 Hz, H-3″,5″), 7.21 (2H, d, *J* = 8.6 Hz, H-3′,5′), 6.71 (1H, d, *J* = 2.2 Hz, H-8), 6.69 (1H, s, H-3), 6.62 (1H, d, *J* = 16.0 Hz, -CH=CHCO-), 6.53 (1H, d, *J* = 2.2 Hz, H-6), 5.28 (2H, s, -OCH_2O-), 3.54 (3H, s, -OCH_3), 2.36 (3H, s, -$COCH_3$). MS (ESI) *m/z:* 519.2(M + H)$^+$

(*E*)-3-(5,7-dihydroxy-4-oxo-4*H*-chromen-2-yl)phenyl 3-(4-acetoxyphenyl)acrylate, 4a

To a solution of **15a** (18 mg, 0.036 mmol) in MeCN (2mL) was added TMSI (9 μL, 0.072 mmol) at -20 ℃ under argon. After 1h stirring, ethyl acetate (2 mL) was added to quench the reaction. The solvent was washed with ammonium thiosulfate and brine, dried over Na_2SO_4, filtered and concentrated *in vacuo*. The crude product was purified by column chromatography (petroleum ether/DCM/THF = 10:10:1) to give **4a** as white solid (12 mg, 72.8%). ^{1}H-NMR (400 MHz, DMSO-*d*$_6$) *δ* 12.83 (1H. s, 5-OH), 9.68 (1H, s, H-7), 7.99 – 7.95 (1H, m, H-6′), 7.92 (1H, d, *J* = 16.0 Hz, -CH=CHCO-), 7.91 (1H, t, *J* = 2.0 Hz, H-2′), 7.83 (2H, d, *J* = 8.7 Hz, H-2″, 6″), 7.65 (1H, t, *J* = 8.0 Hz, H-5′), 7.45 (1H, dd, *J* = 8.1, 2.3 Hz, H-4′), 7.23 (1H, d, *J* = 8.8 Hz, H-3″, 5″), 6.83 (1H, s, H-3), 6.78 (1H, d, *J* = 16.0 Hz, -CH=CHCO-), 6.58 (1H, d, *J* = 2.1 Hz, H-8), 6.26 (1H, d, *J* = 2.1 Hz, H-6), 2.27 (3H, s, -$COCH_3$). ^{13}C-NMR (150 MHz, DMSO-*d*$_6$) *δ* 182.28, 169.43, 165.28, 164.94, 162.48, 161.86, 157.88, 152.85, 151.48, 146.30, 132.73, 131.94, 130.83, 130.47, 126.05, 124.38, 122.96, 120.38, 117.41, 106.23, 104.48, 99.51, 94.68. HR-MS (ESI) calcd for $C_{26}H_{18}O_8$: 458.10017, found [M + H]$^+$: 459.10580

(*E*)-4-(5,7-dihydroxy-4-oxo-4*H*-chromen-2-yl)phenyl 3-(4-acetoxyphenyl)acrylate, 4e

Compound **4e** (6 mg, 65.5%) was synthesized from **15b** (10 mg, 0.02 mmol) according to the procedure used to prepare **4a**. The crude product was purified by column chromatography (DCM/methol = 50:1) to give **4e** as white solids. ^{1}H-NMR (400 MHz, DMSO-*d*$_6$) *δ* 12.76 (1H, s, 5-OH), 8.11 (2H, d, *J* = 8.8 Hz, H-2′, 6′), 7.85 (1H, d, *J* = 16.2 Hz, -CH=CHCO-), 7.84 (2H, d, *J* = 8.6 Hz, H-2″,6″), 7.40 (1H, d, *J* = 8.8 Hz, H-3″,5″), 7.20 (2H, d, *J* = 8.6 Hz, H-3′,5′), 6.92 (1H, s, H-3), 6.85 (1H, d, *J* = 16.0 Hz, -CH=CHCO-), 6.47 (1H, d, *J* = 2.2 Hz, H-8), 6.16 (1H, d, *J* = 2.0 Hz, H-6), 2.25 (3H, s, -$COCH_3$). ^{13}C-NMR (151 MHz, DMSO-*d*$_6$) δ 182.29, 174.75, 169.44, 165.01, 164.87, 162.95, 161.89, 157.88, 153.68, 152.86, 146.45, 131.91, 130.49, 130.07, 128.74, 128.46, 123.13, 122.95, 117.31, 109.98, 105.64, 105.08, 104.37, 101.21, 99.47, 95.44, 94.60, 29.40. HR-MS (ESI) calcd for $C_{26}H_{18}O_8$: 458.10017, found [M + H]$^+$: 459.10583.

(*E*)-3-(5,7-dihydroxy-4-oxo-4*H*-chromen-2-yl)phenyl 3-(4-hydroxyphenyl)acrylate, 4b

To a solution of **4a** (12mg, 0.026mmol) in DCM (1mL) was added K_2CO_3 (3.9 mg, 0.026mmol) in methanol under argon. After 12h stirring, the solvent was concentrated *in vacuo* and purified by column chromatography (DCM/methanol = 40:1) to give **4b** as white solid (4 mg, 35.5%). ^{1}H-NMR (400 MHz, DMSO-*d*$_6$) *δ* 12.75 (1H, s, 5-OH), 10.89 (1H,s, 4″-OH), 10.10 (1H, s, 7-OH), 7.96 (1H, ddd, *J* = 7.9, 1.7, 1.0 Hz, H-6′), 7.90 (1H, t, *J* = 2.0 Hz, H-2′), 7.78 (1H, d, *J* = 15.9 Hz, -CH=CHCO-), 7.64 (2H, d, *J* = 8.7 Hz, H-2″, 6″), 7.60 (1H, t, *J* = 8.0 Hz, H-5′), 7.42 (1H, dd, *J* = 8.1, 2.3 Hz, H-4′), 7.01 (1H, s, H-3), 6.81 (1H, d, *J* = 8.8 Hz, H-3″,5″), 6.64 (1H, d, *J* = 15.9 Hz, -CH=CHCO-), 6.52 (1H, d, *J* = 2.1 Hz, H-8), 6.19 (1H, d, *J* = 2.1 Hz, H-6). ^{13}C-NMR (151 MHz, DMSO-*d*$_6$) δ 182.28, 165.66, 164.93, 161.86, 157.88, 151.48, 147.59, 136.34, 132.67, 131.27 (2), 130.77, 130.07, 125.36, 120.39 (2), 116.34, 113.25, 106.20, 104.47, 99.51, 94.68. HR-MS (ESI) calcd for $C_{24}H_{16}O_7$: 416.08960, found [M + H]$^+$: 417.09625.

(*E*)-4-(5,7-dihydroxy-4-oxo-4*H*-chromen-2-yl)phenyl 3-(4-hydroxyphenyl)acrylate, 4f

Compound **4f** (3 mg, 61.6%) was synthesized from **4e** (5.6 mg, 0.012 mmol) according to the procedure used to prepare **4b**, obtained as white solids. ^{1}H-NMR (400 MHz, DMSO-*d*$_6$) *δ* 8.12 (2H, d, *J* = 8.8 Hz, H-2′,6′), 7.78 (1H, d, *J* = 15.9 Hz, -<u>CH</u>=CHCO-), 7.63 (2H, d, *J* = 8.7 Hz, H-2″,6″), 7.38 (1H, d, *J* = 8.8 Hz, H-3″,5″), 6.96 (1H, s, H-3), 6.80 (2H, d, *J* = 8.6 Hz, H-3′,5′), 6.62 (1H, d, *J* = 16.0 Hz, -CH=<u>CH</u>CO-), 6.50 (1H, d, *J* = 2.0 Hz, H-8), 6.19 (1H, d, *J* = 2.1 Hz, H-6). ^{13}C-NMR (151 MHz, dmso) δ 182.28, 164.85, 161.88, 160.84, 157.88, 147.76,133.34, 131.31, 128.40, 123.15, 116.33, 113.12, 109.55, 107.87, 101.35, 98.86, 94.60, 55.31. HR-MS (ESI) calcd for $C_{24}H_{16}O_7$: 416.08960, found [M + H]$^+$: 417.09620.

5-hydroxy-2-(3-(2-hydroxyethoxy)phenyl)-7-(methoxymethoxy)-4*H*-chromen-4-one, 16a

Ethylene glycol (500 mg, 8.1 mmol) and triethylamine (0.54 mL, 4.0 mmol) were dissolved in 7.5 mL dry CH_2Cl_2, and then TsCl (384 mg, 2.0 mmol) was added into the mixture. The reaction mixture was stirred 12 h at 40°C. The resulting mixture was diluted with CH_2Cl_2, washed with saturated aqueous NH_4Cl , $NaHCO_3$ and NaCl successively, and dried over anhydrous Na_2SO_4. After concentrated *in vacuo*, the residue was purified by column chromatography (CH_2Cl_2/MeOH = 30:1) to give **2-(2-hydroxyethoxy)ethyl 4-methylbenzenesulfonate** (260 mg, 23%) as colorless oil. Potassium carbonate (143 mg, 1.0 mmol) was added to a solution of **14a** (210 mg, 0.7 mmol) and **2-(2-hydroxyethoxy)ethyl 4-methylbenzenesulfonate** (223 mg, 1.0 mmol) in CH_3CN (4 mL) at room temperature. The reaction mixture was then refluxed overnight. The resulting mixture was poured into ice-water, adjusted to neutral pH with aqueous HCl (5%), and extracted with EtOAc. The organic layer was washed with brine, dried over Na_2SO_4, filtered and concentrated *in vacuo*. The residue was purified by column chromatography (petroleum ether /acetone = 5:1) to give **16a** as light yellow solid (105 mg, 44.5%). ^{1}H-NMR (500 MHz, Acetone-*d*$_6$) *δ* 12.85 (1H, s, 5-OH), 7.67 (1H, d, *J* = 7.8 Hz, H-6′), 7.65 (1H, t, J=2.3Hz, H-2′), 7.52 (1H, t, *J* = 8.0 Hz, H-5′), 7.22 (1H, dd, *J* = 8.3, 2.5 Hz, H-4′), 6.87 (1H, s, H-3), 6.84 (1H, d, *J* = 2.2 Hz, H-8), 6.46 (1H, d, *J* = 2.1 Hz, H-6), 5.36 (2H, s, -OCH_2O-), 4.24 – 4.22 (2H, m, -OCH_2-), 3.96 – 3.93 (2H, m, -OCH_2-), 3.51 (3H, s, -OCH_3). MS (ESI) *m*/*z:* 375.1(M + H)$^+$

5-hydroxy-2-(4-(2-hydroxyethoxy)phenyl)-7-(methoxymethoxy)-4*H*-chromen-4-one, 16b

Compound **16b** (18 mg, 75.0%) was synthesized from **14b** (21 mg, 0.067 mmol) according to the procedure used to prepare **16a**, obtained as light-yellow solids. ^{1}H-NMR (500 MHz, Acetone-*d*$_6$) *δ* 12.85 (1H, s, 5-OH), 7.76 (2H, d, *J* = 8.6 Hz, H-2′,6′), 7.22 (2H, d, *J* = 8.6 Hz, H-3′,5′), , 6.87 (1H, s, H-3), 6.84 (1H, d, *J* = 2.2 Hz, H-8), 6.46 (1H, d, *J* = 2.1 Hz, H-6), 5.36 (2H, s, -OCH_2O-), 4.24 – 4.22 (2H, m, -OCH_2-), 3.96 – 3.93 (2H, m, -OCH_2-), 3.51 (3H, s, -OCH_3). MS (ESI) *m*/*z:* 359.1(M + H)$^+$

(*E*)-2-(3-(5-hydroxy-7-(methoxymethoxy)-4-oxo-4*H*-chromen-2-yl)phenoxy)ethyl 3-(4-acetoxyphenyl)acrylate, 17a

To a solution of **4-acetoxycinnamic acid** (41mg, 0.2 mmol) in THF (1 mL) were added EDCI (38 mg, 0.2 mmol), DMAP (6 mg, 0.05 mmol) and Et_3N (45 μL, 0.3 mmol). Then **16a** (24 mg, 0.066 mmol) in THF (1 mL) was added under nitrogen. The solution was stirred at r.t. for 12 h. The organic layer was concentrated *in vacuo*. The residue was purified by column chromatography (petroleum ether/EtOAc = 3:1) to give **17a** as white solid (12 mg, 33.3%). ^{1}H-NMR (500 MHz, Acetone-*d*$_6$) *δ* 12.84 (1H, s, H-5), 7.74 -7.70 (5H, m, -<u>CH</u>=CHCO- and H-2″, 6″ and H-2′, 6′), 7.55 (1H, t, *J* = 8.0 Hz, H-5′), 7.27 (1H, dd, *J* = 8.3, 2.5 Hz, H-4′), 7.20 (2H, d, *J* = 8.7 Hz, H-3″,5″), 6.90 (1H, s, H-3), 6.85 (1H, d, *J* = 2.2 Hz, H-8), 6.59 (1H, d, *J* = 16.0 Hz, -CH=<u>CH</u>CO-), 6.46 (1H, d, *J* = 2.2 Hz, H-6), 5.36 (2H, s, -OCH_2O-), 4.64 – 4.59 (2H, m, -OCH_2-), 4.51 – 4.48 (2H, m, -OCH_2-), 3.50 (3H, s, -OCH_3), 2.29 (3H, s, -$COCH_3$). MS (ESI) *m*/*z:* 563.2(M + H)$^+$

(*E*)-2-(4-(5-hydroxy-7-(methoxymethoxy)-4-oxo-4*H*-chromen-2-yl)phenoxy)ethyl 3-(4-acetoxyphenyl)acrylate, 17b

Compound **17b** (6 mg, 33.3%) was synthesized from **16b** (12 mg, 0.033 mmol) according to the procedure used to prepare **17a**, obtained as white solids. ^{1}H-NMR (500 MHz, Acetone-*d*$_6$) *δ* 12.99 (1H, s, 5-OH), 9.63 (1H, s, 7-OH), 8.06 (2H, d, *J* = 8.9Hz, H-2′,6′), 7.76 (2H, d, *J* = 8.6 Hz, H-2″,6′), 7.75 (1H, d, *J* = 16.2 Hz, -<u>CH</u>=CHCO-), 7.22 (2H, d, *J* = 8.5Hz, H-3′,5′), 7.22 (2H, d, *J* = 8.8 Hz, H-3″,5″), 6.71

(1H, s, H-3), 6.58 (1H, d, *J* = 15.8 Hz, -CH=CHCO-), 6.57 (1H, d, *J* = 2.1 Hz, H-8), 6.28 (1H, d, *J* = 2.1 Hz, H-6), 5.36 (2H, s, -OCH_2O-), 4.62 – 4.59 (2H, m, -OCH_2-), 4.49 – 4.46 (2H, m, -OCH_2-), 3.48 (3H, s, -OCH_3), 2.29 (3H, s, -CH_3). MS (ESI) *m/z:* 563.2(M + H)$^+$

(*E*)-2-(3-(5,7-dihydroxy-4-oxo-4*H*-chromen-2-yl)phenoxy)ethyl 3-(4-acetoxyphenyl)acrylate, 4c

Compound **4c** (10.3 mg, 93.3%) was synthesized from **17a** (12 mg, 0.02 mmol) according to the procedure used to prepare **4a**, obtained as white solids. ^{1}H-NMR (500 MHz, Acetone-d_6) δ 12.88 (1H, s, 5-OH), 9.60 (1H, s, H-7), 7.76 – 7.66 (5H, m, -CH=CHCO- and H-2″, 6″ and H-2′, 6′), 7.54 (1H, t, *J* = 7.9 Hz, H-5′), 7.25 (1H, dd, *J* = 8.3, 2.5 Hz, H-4′), 7.20 (2H, d, *J* = 8.7 Hz, H-3″, 5″), 6.83 (1H, s, H-3), 6.61 (1H, d, *J* = 2.2 Hz, H-8), 6.58 (1H, d, *J* = 16.0 Hz, -CH=CHCO-), 6.30 (1H, d, *J* = 2.0 Hz, H-6), 4.63 – 4.59 (2H, m, -OCH_2-), 4.51 – 4.47 (2H, m, -OCH_2-), 2.29 (3H, s, -$COCH_3$). ^{13}C-NMR (125 MHz, Acetone-d_6) δ 182.45, 168.65, 166.23, 164.45, 163.71, 162.71, 159.59, 158.24, 152.87, 144.08, 133.09, 132.14, 130.49, 129.85, 129.47, 122.48, 119.24, 118.58, 118.03, 112.59, 105.85, 104.95, 99.22, 94.26, 66.69, 62.91, 20.21. HR-MS (ESI) calcd for $C_{28}H_{22}O_9$: 502.12638, found [M + H]$^+$: 503.13263.

(*E*)-2-(4-(5,7-dihydroxy-4-oxo-4*H*-chromen-2-yl)phenoxy)ethyl 3-(4-acetoxyphenyl)acrylate, 4g

Compound **4g** (3.4 mg, 65.5%) was synthesized from **17a** (6 mg, 0.01 mmol) according to the procedure used to prepare **4a**. The crude product was purified by column chromatography (DCM/methol = 50:1) to give **4g** as white solids. ^{1}H-NMR (500 MHz, Acetone-d_6) δ 12.99 (1H, s, 5-OH), 9.62 (1H, s, 7-OH), 8.06 (2H, d, *J* = 8.9Hz, H-2′, 6′), 7.76 (2H, d, *J* = 8.6 Hz, H-2″, 6″), 7.73 (1H, d, *J* = 16.2 Hz, -CH=CHCO-), 7.22 (2H, d, *J* = 8.6 Hz, H-3′,5′), 7.20 (2H, d, *J* = 8.8 Hz, H-3″,5″), 6.71 (1H, s, H-3), 6.58 (1H, d, *J* = 15.8 Hz, -CH=CHCO-), 6.57 (1H, d, *J* = 2.1 Hz, H-8), 6.28 (1H, d, *J* = 2.1 Hz, H-6), 4.62 – 4.59 (2H, m, -OCH_2-), 4.49 – 4.46 (2H, m, -OCH_2-), 2.29 (3H, s, -CH_3). ^{13}C-NMR (150 MHz, acetone-d_6) δ 182.16, 168.56, 166.06, 164.19, 163.74, 162.42, 161.80, 157.90, 152.63, 143.94, 143.40, 131.90, 129.64, 129.34, 128.23, 124.82, 123.81, 122.34, 117.71, 115.12, 104.41, 103.75, 98.88, 93.88, 66.40, 62.58 , 20.05. HR-MS (ESI) calcd for $C_{28}H_{22}O_9$: 502.12638, found [M + H]$^+$: 503.10266.

(*E*)-2-(3-(5,7-dihydroxy-4-oxo-4*H*-chromen-2-yl)phenoxy)ethyl 3-(4-hydroxyphenyl)acrylate, 4d

Compound **4d** (5.4 mg, 43.5%) was synthesized from **4c** (12 mg, 0.026 mmol) according to the procedure used to prepare **4b**. The crude product was purified by column chromatography (DCM/methol = 40:1) to give **4d** as white solids. ^{1}H-NMR (500 MHz, Acetone-d_6) δ 12.88 (1H, s, 5-OH), 9.78 (1H, s, H-7), 7.76 – 7.66 (5H, m, -CH=CHCO- and H-2″, 6″ and H-2′, 6′), 7.54 (1H, t, *J* = 7.9 Hz, H-5′), 7.25 (1H, dd, *J* = 8.3, 2.5 Hz, H-4′), 7.21 (2H, d, *J* = 8.7 Hz, H-3″, 5″), 6.83 (1H, s, H-3), 6.61 (1H, d, *J* = 2.2 Hz, H-8), 6.58 (1H, d, *J* = 16.0 Hz, -CH=CHCO-), 6.30 (1H, d, *J* = 2.0 Hz, H-6), 4.63 – 4.59 (2H, m, -OCH_2-), 4.54 – 4.49 (2H, m, -OCH_2-). ^{13}C-NMR (125 MHz, Acetone-d_6) δ 182.45, 166.65, 164.42, 163.72, 162.71, 159.98, 159.62, 158.23, 145.16, 133.07, 130.47, 130.26, 126.26, 119.21, 118.60, 116.01, 114.51, 112.57, 105.84, 104.96, 99.21, 94.25, 66.76, 62.65. HR-MS (ESI) calcd for $C_{26}H_{20}O_8$: 460.11582, found [M + H]$^+$: 461.12262.

(*E*)-2-(4-(5,7-dihydroxy-4-oxo-4*H*-chromen-2-yl)phenoxy)ethyl 3-(4-hydroxyphenyl)acrylate, 4h

Compound **4h** (2.2 mg, 57.6%) was synthesized from **4g** (4 mg, 0.008 mmol) according to the procedure used to prepare **4b**. The crude product was purified by column chromatography (DCM/methol = 40:1) to give **4h** as white solids. ^{1}H-NMR (400 MHz, DMSO-d_6) δ 12.88 (1H, s, 5-OH), 10.82 (1H, s, 4′-OH), 9.98 (1H, s, 7-OH), 8.01 (2H, d, *J* = 9.0 Hz, H-3″, 5″), 7.52 (1H, d, *J* = 16.0 Hz, -CH=CHCO-), 7.52 (2H, d, *J* = 8.4 Hz, H-2′,6′), 7.13 (2H, d, *J* = 9.0 Hz, H-3′,5′), 6.85 (1H, s, H-3), 6.74 (1H, d, *J* = 8.8 Hz, H-3″, 5″), 6.47 (1H, d, *J* = 2.1 Hz, H-8), 6.41 (1H, d, *J* = 15.9 Hz, -CH=CHCO-), 6.17 (1H, d, *J* = 2.1 Hz, H-6), 4.48 – 4.41 (2H, m, -OCH_2-), 4.38 – 4.32 (2H, m, -OCH_2-). ^{13}C-NMR (151 MHz, DMSO-d_6) δ 182.21, 166.98, 164.66, 163.62, 161.87, 160.36, 157.78, 145.69, 130.86, 128.81 (2), 127.05 (2), 125.42 (2), 123.56, 116.18 (2), 115.58, 114.14, 104.08, 99.33, 94.48, 66.72, 62.76. HR-MS (ESI) calcd for $C_{26}H_{20}O_8$: 460.11582, found [M + H]$^+$: 461.12263.

1.1.2. Synthesis of compound **4i**

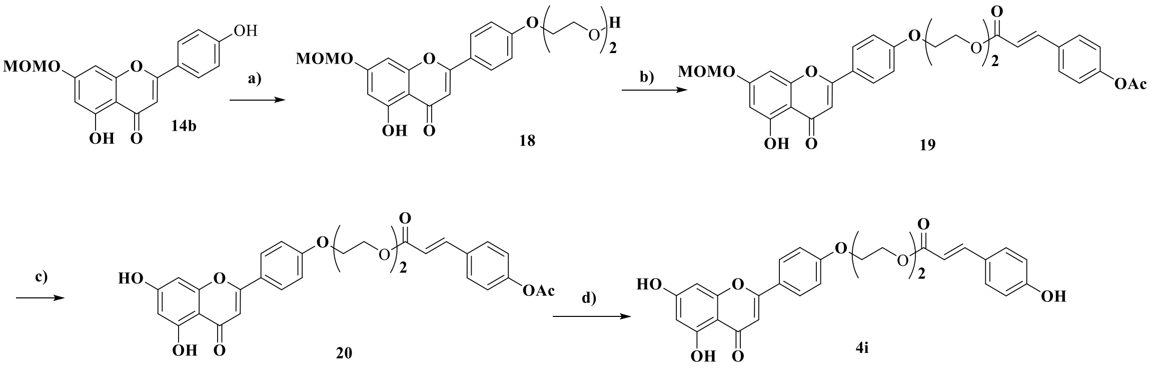

a, 6, K_2CO_3, MeCN, 80°C, 12h; b, 4-acetylcoumaric acid, EDCI, DMAP, TEA,THF, r.t. 24h; c, TMSI, MeCN, -20°C, 1h; d, K_2CO_3, MeOH, DCM, r.t., 12h.

Scheme S2. Synthesis of compound 4i.

2-(2-(4-(5,7-dihydroxy-4-oxo-4*H*-chromen-2-yl)phenoxy)ethoxy)ethyl (E)-3-(4-hydroxyphenyl)acrylate, 4i. Compound **4i** (2.0 mg, 20.4% for 4 steps) was synthesized from **14b** (41 mg, 0.13 mmol) according to the procedure used to prepare **4b,** obtained as white solids.

5-hydroxy-2-(4-(2-(2-hydroxyethoxy)ethoxy)phenyl)-7-(methoxymethoxy)-4*H*-chromen-4-one, 18. ^{1}H-NMR (500 MHz, Acetone-d_6) *δ* 12.85 (1H, s, 5-OH), 7.76 (2H, d, *J* = 8.6 Hz, H-2′,6′), 7.22 (2H, d, *J* = 8.6 Hz, H-3′,5′), , 6.87 (1H, s, H-3), 6.84 (1H, d, *J* = 2.2 Hz, H-8), 6.46 (1H, d, *J* = 2.1 Hz, H-6), 5.36 (2H, s, -OCH$_2$O-), 4.30(2H, m, -OCH$_2$-), 4.24 (2H, m, -OCH$_2$-), 3.84 (2H, m, -OCH$_2$-), 3.78 (2H, m, -OCH$_2$-),3.51 (3H, s, -OCH$_3$). MS (ESI) *m*/*z:* 403.1(M + H)$^+$

2-(2-(4-(5-hydroxy-7-(methoxymethoxy)-4-oxo-4*H*-chromen-2-yl)phenoxy)ethoxy)ethyl (*E*)-3-(4-acetoxyphenyl)acrylate, 19. ^{1}H-NMR (500 MHz, Acetone-d_6) *δ* 12.99 (1H, s, 5-OH), 9.63 (1H, s, 7-OH), 8.06 (2H, d, *J* = 8.9Hz, H-2′,6′), 7.76 (2H, d, *J* = 8.6 Hz, H-2″,6′), 7.75 (1H, d, *J* = 16.2 Hz, -<u>CH</u>=CHCO-), 7.22 (2H, d, *J* = 8.5Hz, H-3′,5′), 7.22 (2H, d, *J* = 8.8 Hz, H-3″,5″), 6.71 (1H, s, H-3), 6.58 (1H, d, *J* = 15.8 Hz, -CH=<u>CH</u>CO-), 6.57 (1H, d, *J* = 2.1 Hz, H-8), 6.28 (1H, d, *J* = 2.1 Hz, H-6), 5.36 (2H, s, -OCH$_2$O-), 4.30(2H, m, -OCH$_2$-), 4.24 (2H, m, -OCH$_2$-), 3.84 (2H, m, -OCH$_2$-), 3.78 (2H, m, -OCH$_2$-), 3.48 (3H, s, -OCH$_3$), 2.29 (3H, s, -CH$_3$). MS (ESI) *m*/*z:* 591.2(M + H)$^+$

2-(2-(4-(5,7-dihydroxy-4-oxo-4*H*-chromen-2-yl)phenoxy)ethoxy)ethyl (E)-3-(4-acetoxyphenyl)acrylate, 20. ^{1}H-NMR (500 MHz, Acetone-d_6) *δ* 12.99 (1H, s, 5-OH), 9.62 (1H, s, 7-OH), 8.06 (2H, d, *J* = 8.9Hz, H-2′, 6′), 7.76 (2H, d, *J* = 8.6 Hz, H-2″, 6″), 7.73 (1H, d, *J* = 16.2 Hz, -<u>CH</u>=CHCO-), 7.22 (2H, d, *J* = 8.6 Hz, H-3′,5′), 7.20 (2H, d, *J* = 8.8 Hz, H-3″,5″), 6.71 (1H, s, H-3), 6.58 (1H, d, *J* = 15.8 Hz, -CH=<u>CH</u>CO-), 6.57 (1H, d, *J* = 2.1 Hz, H-8), 6.28 (1H, d, *J* = 2.1 Hz, H-6), 4.30(2H, m, -OCH$_2$-), 4.24 (2H, m, -OCH$_2$-), 3.84 (2H, m, -OCH$_2$-), 3.78 (2H, m, -OCH$_2$-), 2.29 (3H, s, -CH$_3$). MS (ESI) *m*/*z:* 565.2 (M + H)$^+$

2-(2-(4-(5,7-dihydroxy-4-oxo-4*H*-chromen-2-yl)phenoxy)ethoxy)ethyl (E)-3-(4-hydroxyphenyl)acrylate, 4i. ^{1}H-NMR (400 MHz, DMSO-d_6) δ 12.92 (1H, s, 5-OH), 10.84 (1H, s, 4′-OH), 9.99 (1H, s, 7-OH), 8.01 (2H, d, *J* = 9.0 Hz, H-3″, 5″), 7.52 (1H, d, *J* = 16.0 Hz, -<u>CH</u>=CHCO-), 7.52 (2H, d, *J* = 8.4 Hz, H-2′,6′), 7.13 (2H, d, *J* = 9.0 Hz, H-3′,5′), 6.86 (1H, s, H-3), 6.77 (1H, d, *J* = 8.8 Hz, H-3″, 5″), 6.50 (1H, d, *J* = 2.1 Hz, H-8), 6.41 (1H, d, *J* = 15.9 Hz, -CH=<u>CH</u>CO-), 6.20 (1H, d, *J* = 2.1 Hz, H-6), 4.28 – 4.22 (4H, m, 2×-OCH$_2$-), 3.84 – 3.74 (4H, m, 2×-OCH$_2$-).^{13}C-NMR(150MHz, DMSO-d$_6$) δ 182.20, 167.02, 164.63, 163.66, 161.97, 161.86, 160.29, 157.76, 145.37, 130.78, 128.74, 125.43, 123.29, 116.15, 115.50, 114.35, 109.99, 104.19, 103.96, 100.88, 99.31, 98.66, 94.47, 83.12, 79.61, 79.53, 69.38, 69.17, 69.00, 68.07, 67.98, 66.10. HR-MS (ESI) calcd for $C_{28}H_{24}O_9$: 504.14203, found [M + H]$^+$: 505.14905.

1.1.3. Synthesis of compound **4k**

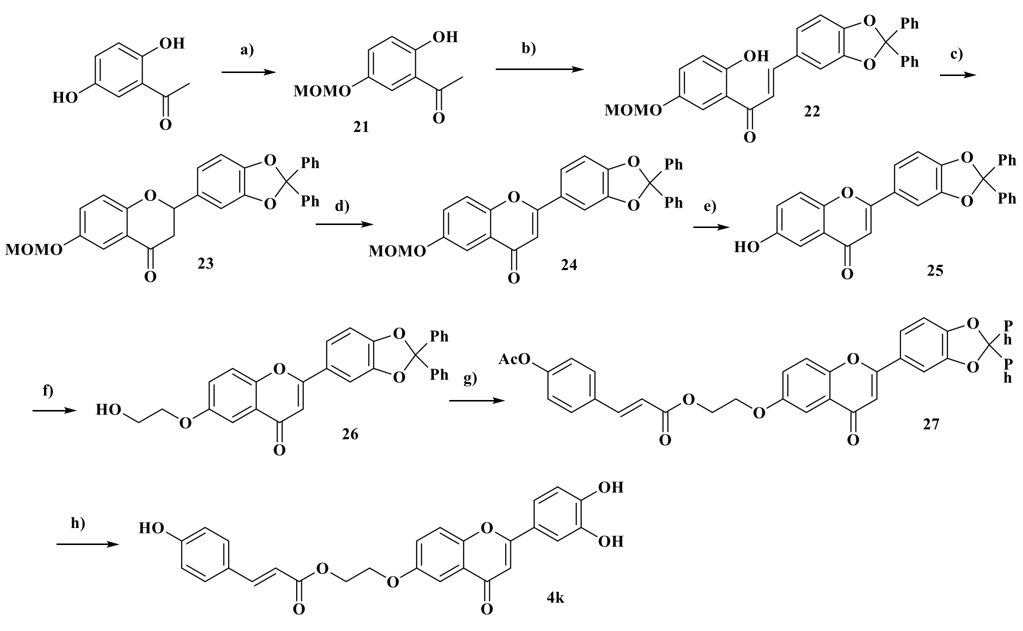

a, MOMCl, DIEA, DCM, -10°C to 0°C, 1h; b, 20% KOH aq, EtOH, r.t. 1h; c, CH_3COONa, H_2O, EtOH, 80°C, 12h; d, I_2, Py, 100°C, 12h; e, TMSI, MeCN, N_2, -20°C , 1h; f, $TsOCH_2CH_2OH$,K_2CO_3, MeCN, 80°C, 12h; g, 4-acetylcoumaric acid, EDCI, DMAP, TEA,THF, r.t. 24h; h, CH_3COOH/HCl, Acetone, r.t. 24h.

Scheme S3. Synthesis of compound **4k**.

1-(2-hydroxy-5-(methoxymethoxy)phenyl)ethan-1-one, 21

Compound **21** (350 mg, 91.1%) was synthesized from **1-(2,5-dihydroxyphenyl)ethan-1-one** (300 mg, 2 mmol) according to the procedure used to prepare **9**, obtained as white solids. ^{1}H-NMR (400 MHz, $CDCl_3$) δ 11.90 (1H, s, 2-OH), 7.38 (1H, d, *J* = 2.9 Hz, H-6), 7.21 (1H, dd, *J* = 9.0, 2.9 Hz, H-4), 6.91 (1H, d, *J* = 9.0 Hz, H-3), 5.11 (2H, s, $-OCH_2O-$), 3.49 (3H, s, $-OCH_3$), 2.60 (3H, s, $-CH_3$). MS (ESI) *m*/*z:* 197.1 $(M + H)^+$. The spectroscopic data of **21** were consistent with the reference. [S4]

(E)-3-(2,2-diphenylbenzo[*d*][1,3]dioxol-5-yl)-1-(2-hydroxy-5-(methoxymethoxy)phenyl)prop-2-en-1-one, 22

Dichlorodiphenylmethane (1.3mL, 7.5 mmol) and K_2CO_3 (1.38g, 10mmol) were added to a stirred mixture of 3,4-dihydroxybenzaldehyde (690 mg, 5 mmol) in DMF (1.4 mL). After stirring at r.t. for 2h, water (40 mL) was added and extracted with ethyl acetate (40 mL*2). The organic layer was gathered dried over Na_2SO_4, filtered and concentrated *in vacuo*. The crude product was purified by column chromatography (PE/acetone = 6:1) to give **2,2-diphenylbenzo[*d*][1,3]dioxole-5-carbaldehyde** as yellow solids (901 mg, 60.1%). ^{1}H-NMR (400 MHz, $CDCl_3$) δ 9.82 (1H, s, -CHO), 7.61 – 7.56 (4H, m, H-*o* of diphenylmethane), 7.55 (1H, dd, *J* = 8.0, 1.6 Hz, H-6), 7.48 – 7.39 (7H, m, H-*m,p* of diphenylmethane and H-2), 7.15 (1H, d, *J* = 8.0 Hz, H-5). MS (ESI) *m*/*z:* 303.1 $(M + H)^+$

Compound **22** (150 mg, 52.0%) was synthesized from **21** (110 mg, 0.6 mmol) and **2,2-diphenylbenzo[*d*][1,3]dioxole-5-carbaldehyde** (180mg, 0.6mmol) according to the procedure used to prepare **11a**, obtained as yellow solid. ^{1}H-NMR (400 MHz, $CDCl_3$) δ 7.88 – 7.84 (3H, m, H-2′,H-3 and H-5′), 7.65 (1H, d, *J* = 1.6 Hz, H-6), 7.61 – 7.57 (4H, m, H-*o* of diphenylmethane), 7.48 – 7.41 (6H, m, H-*m,p* of diphenylmethane), 7.37 (1H, dd, *J* = 8.1, 1.7 Hz, H-6′), 7.29 (1H, dd, *J* = 9.0, 2.9 Hz, H-4), 7.05 (1H, d, *J* = 8.1 Hz, -<u>CH</u>=CHCO-), 6.90 (1H, d, *J* = 9.0 Hz, -CH=<u>CH</u>CO-), 5.17 (2H, s, $-OCH_2O-$), 3.43 (3H, s, $-CH_3$). MS (ESI) *m*/*z:* 481.1 $(M + H)^+$

2-(2,2-diphenylbenzo[*d*][1,3]dioxol-5-yl)-6-(methoxymethoxy)chroman-4-one，23

Compound **23** (22 mg, 46.2%) was synthesized from **22** (40 mg, 0.1 mmol) and according to the procedure used to prepare **12a**, obtained as white solid. ^{1}H-NMR (400 MHz, Acetone-*d*$_6$) δ 7.61 – 7.57 (4H, m, H-*o* of diphenylmethane), 7.46 – 7.38 (7H, m, H-*m,p* of diphenylmethane and H-8), 7.23 (1H, dd, *J* = 9.0, 3.1 Hz, H-7), 7.20 (1H, d, *J* = 1.8 Hz, H-5′), 7.07 – 7.04 (1H, m, H-6′), 6.99 (1H, s, H-5), 6.97 (1H, s, H-2′), 5.46 (1H, dd, *J* = 13.2, 2.8 Hz, H-2), 5.15 (2H, s, -OCH$_2$O-), 3.40 (3H, s, -CH$_3$), 3.10 (1H, dd, *J* = 16.8, 13.2 Hz, H-3a), 2.76 (1H, dd, *J* = 16.8, 2.9 Hz, H-3b). MS (ESI) *m/z:* 481.1 (M + H)$^+$

2-(2,2-diphenylbenzo[*d*][1,3]dioxol-5-yl)-6-(methoxymethoxy)-4*H*-chromen-4-one, 24

Compound **24** (16 mg, 79.7%) was synthesized from **23** (20 mg, 0.042 mmol) and according to the procedure used to prepare **13a**, obtained as yellow solid. ^{1}H-NMR (400 MHz, Acetone-*d*$_6$) δ 7.76 –7.73 (4H, m, H-6′, H-5′, H-2′ and H-5), 7.68 – 7.66 (4H, m, H-*o* of diphenylmethane), 7.53 – 7.48 (6H, m, H-*m, p* of diphenylmethane and H-7), 7.21 (1H, d, *J* = 8.3 Hz, H-8), 6.80 (1H, s, H-3), 5.35 (2H, s, -OCH$_2$O-), 3.51 (3H, s, -OCH$_3$) . MS (ESI) *m/z:* 479.1 (M + H)$^+$

2-(2,2-diphenylbenzo[*d*][1,3]dioxol-5-yl)-6-hydroxy-4*H*-chromen-4-one, 25

The starting material **24** (20 mg, 0.042 mmol) was dissolved in MeCN (1 mL) and TMSI (30 μL, 0.4 mmol) was added at -20°C under Ar. After 1h stirring, water (4 mL) was added slowly and the suspension heated to 0°C and extracted with ethyl acetate (10 ml). The organic phase was merged, then washed with water and brine, dried over Na$_2$SO$_4$, filtered and concentrated *in vacuo*. The residue was purified by column chromatography (petroleum ether/acetone = 5:1) to give **25** as yellow solids (17 mg, 93.2%). ^{1}H-NMR (400 MHz, Acetone-*d*$_6$) δ 7.75 (2H, m, H-6′ and H-5′), 7.68 (1H, d, *J* = 2.2 Hz, H-2′), 7.66 (1H, s, H-5), 7.61 – 7.59 (4H, m, H-*o* of diphenylmethane), 7.40 – 7.38 (6H, m, H-*m,p* of diphenylmethane), 7.27 (1H, dd, *J* = 9.1, 3.1 Hz, H-7), 7.14 (1H, d, *J* = 8.3 Hz, H-8), 6.69 (1H, s, H-3). MS (ESI) *m/z:* 435.1 (M + H)$^+$

2-(2,2-diphenylbenzo[*d*][1,3]dioxol-5-yl)-6-(2-hydroxyethoxy)-4*H*-chromen-4-one, 26

Compound **26** (105 mg, 53.8%) was synthesized from **25** (170 mg, 0.39 mmol) and according to the procedure used to prepare **16a**, obtained as yellow solid. ^{1}H-NMR (400 MHz, Acetone-*d*$_6$) δ 7.59 – 7.57 (4H, m, H-*o* of diphenylmethane), 7.49 (2H, m, H-6′ and H-5′), 7.43 (1H, d, *J* = 2.2 Hz, H-2′), 7.40 – 7.38 (6H, m, H-*m, p* of diphenylmethane), 7.30 (1H, dd, *J* = 9.1, 3.1 Hz, H-7), 6.99 (1H, d, *J* = 8.3 Hz, H-8), 6.97 (1H, s, H-3), 6.74 (1H, s, H-5), 4.19 (2H, m, -OCH$_2$-), 4.01 (2H, m, -OCH$_2$-). MS (ESI) *m/z:* 479.1 (M + H)$^+$

2-((2-(2,2-diphenylbenzo[*d*][1,3]dioxol-5-yl)-4-oxo-4*H*-chromen-6-yl)oxy)ethyl (*E*)-3-(4-acetoxyphenyl)acrylate, 27

Compound **27** (26 mg, 19.5%) was synthesized from **26** (100 mg, 0.2 mmol) according to the procedure used to prepare **17a**. The crude product was purified by column chromatography (PE/acetone/THF = 6:1:1) to give **27** as yellow solids. ^{1}H-NMR (400 MHz, Acetone-*d*$_6$) δ 7.69 (1H, d, *J* = 16.0 Hz, -<u>CH</u>=CHCO-), δ 7.61 – 7.55 (4H, m, H-*o* of diphenylmethane), 7.53 (2H, d, *J* = 8.7 Hz, H-2″,6″), 7.49 (2H, m, H-6′ and H-5′), 7.43 (1H, d, *J* = 2.2 Hz, H-2′), 7.44 – 7.42 (6H, m, H-*m, p* of diphenylmethane), 7.30 (1H, dd, *J* = 9.1, 3.1 Hz, H-7), 7.11 (2H, d, *J* = 8.6 Hz, H-3″,5″), 6.99 (1H, d, *J* = 8.3 Hz, H-8), 6.97 (1H, s, H-3), 6.70 (1H, s, H-5), 6.43 (1H, d, *J* = 16.0 Hz, -CH=<u>CH</u>CO-), 4.44 (2H, m, -OCH$_2$-), 4.27 (2H, m, -OCH$_2$-), 2.11 (3H, s, -CH$_3$). MS (ESI) *m/z:* 667.2 (M + H)$^+$

2-((2-(3,4-dihydroxyphenyl)-4-oxo-4*H*-chromen-6-yl)oxy)ethyl-(*E*)-3-(4-hydroxyphenyl)acrylate, 4k

27 (20 mg, 0.03 mmol) was dissolved in a solution of acetone (0.2 mL) and 38% HCl/AcOH (v/v = 1:9, 2 mL). The mixture was stirred at room temperature for 24 h. The resulting mixture was poured into ice-water and extracted with EtOAc. The organic layer was washed with brine, dried over anhydrous Na$_2$SO$_4$ and concentrated *in vacuo*. The residue was purified by column chromatography (PE/acetone = 3:1) to give **4k** as yellow solid (5.2 mg, 37.6 %). ^{1}H-NMR (400 MHz, DMSO-*d*$_6$) δ 10.00 (1H, s, 3′-OH), 9.85 (1H, s, 4′-OH), 9.36 (1H, s, 4″-OH), 7.65 (1H, d, *J* = 8.9 Hz, H-7),7.53 (1H, d, *J* = 16.0

Hz, -<u>CH</u>=CHCO-), 7.51 (2H, d, *J* = 8.7 Hz, H-2″,6″), 7.49 (2H, m, H-6′ and H-5′), 7.43 (1H, d, *J* = 2.2 Hz, H-2′), 6.99 (1H, d, *J* = 8.3 Hz, H-8), 6.74 (2H, d, *J* = 8.6 Hz, H-3″,5″), 6.70 (1H, s, H-5), 6.69 (1H, s, H-3), 6.41 (1H, d, *J* = 16.0 Hz, -CH=<u>CH</u>CO-), 4.46 (2H, m, -OCH_2-), 4.35 (2H, m, -OCH_2-). ^{13}C-NMR (151 MHz, DMSO-*d*$_6$) δ 176.92, 166.99, 163.47, 160.34, 155.88, 150.78, 149.79, 146.15, 145.63, 133.06, 130.85, 130.07, 125.43, 124.44, 123.74, 122.42, 120.40, 119.16, 116.41, 116.18, 115.33, 114.19, 113.75, 106.23, 104.58, 67.00, 62.87. HR-MS (ESI) calcd for $C_{26}H_{20}O_8$: 460.11582, found $[M + H]^+$: 461.12219.

1.1.4. Synthesis of compound **4l**

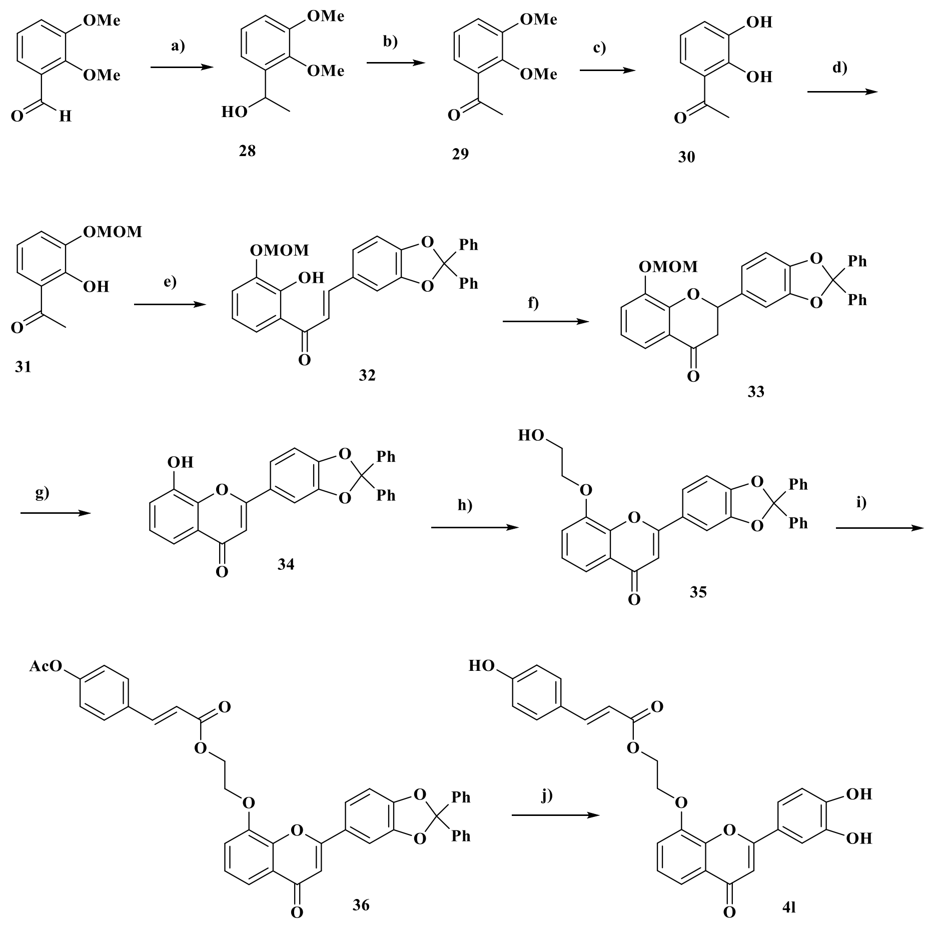

a, CH_3MgBr,THF, r.t.1h; b, PCC, silica gel, DCM, r.t.4h; c,BBr_3, DCM, -30°C, 2h; d,MOMCl, DIEA, DCM, -10°C to 0°C, 1h; e, 20% KOH aq, EtOH, r.t. 1h; f, CH_3COONa, H_2O, EtOH, 80°C, 12h; g, I_2, Py, 100°C, 12h; h, $TsOCH_2CH_2OH$,K_2CO_3, MeCN, 80°C, 12h; i, 4-acetylcoumaric acid, EDCI, DMAP, TEA,THF, r.t. 24h; j, CH_3COOH/HCl, Acetone, r.t. 24h.

.

Scheme S4. Synthesis of compound 4l.

1-(2,3-dimethoxyphenyl)ethan-1-ol, 28

2,3-Dimethoxybenzaldehyde (500 mg, 3 mmol) was added a diethyl ether solution of MeMgBr (2 mL, 3M) under argon at 0 ℃. After stirring for 4 h at room temperature, the reaction was quenched by dropwise added NH_4Cl agueous solution (4 mL) and extracted with EtOAc. The organic layer was dried over Na_2SO_4, filtered and concentrated *in vacuo*. The residue was purified by column chromatography (petroleum ether /acetone = 8:1) to give **28** as light white solid (440 mg, 81.5%). ^{1}H-NMR (400 MHz, $CDCl_3$) δ 7.07 (1H, ddd, *J* = 7.9, 1.6, 0.6 Hz, H-4), 7.00 (1H, t, *J* = 7.9, H-5), 6.86 (1H, dd, *J* = 8.0, 1.6 Hz, H-6), 5.12(1H, m, -CH-), 3.80(3H, s, -CH_3), 3.76(3H, s, -CH_3), 1.32 (3H, d, *J* = 6.4, -CCH_3), MS (ESI) *m*/*z:* 183.1 $(M + H)^+$. The spectroscopic data of **28** were consistent with the reference. [S5]

1-(2,3-dimethoxyphenyl)ethan-1-one，29

Silica gel (1.8 g) and PCC (1.8 g, 10 mmol) were added to a solution of **28** (440 mg, 2.4 mmol) in DCM (20 mL) at room temperature. The reaction mixture was stirred 4h at room temperature. After concentrated in vacuo, the residue was purified by column chromatography (petroleum ether /acetone = 15:1) to give **29** (390mg, 88.6%) as white solid. ^{1}H-NMR (400 MHz, $CDCl_3$) δ 7.18 (1H, dd, *J* = 7.9, 1.6, H-4), 7.12 (1H, m, H-5), 7.08 (1H, m, H-6), 3.88(3H, s, -OCH_3), 3.87(3H, s, -OCH_3), 2.51 (3H, s, -CCH_3), MS (ESI) *m/z:* 181.1 $(M + H)^+$. The spectroscopic data of **29** were consistent with the reference. [S5]

1-(2,3-dihydroxyphenyl)ethan-1-one, 30

BBr_3 (600 μL, 6.2 mmol) was added to a DCM solution of **29** (383 mg, 3.1 mmol) at -30 ℃. After stirring for 2 h, the reaction was quenched by dropwise added saturated $NaHCO_3$ agueous solution (20 mL) and extracted with DCM. The organic layer was dried over Na_2SO_4, filtered and concentrated *in vacuo* to give **30** as light white solid (302 mg, 91.5%). ^{1}H-NMR (400 MHz, Acetone-d_6) δ 12.40 (1H, s, 2-OH), 7.88 (1H, s, 3-OH), 7.38 (1H, dd, *J* = 8.1, 1.5 Hz, H-4), 7.06 (1H, m, H-6), 6.79 (1H, m H-5), 2.62 (3H, s, -CH_3). MS (ESI) *m/z:* 153.1 $(M + H)^+$. The spectroscopic data of **30** were consistent with the reference. [S5]

1-(2-hydroxy-3-(methoxymethoxy)phenyl)ethan-1-one, 31

Compound **31** (350 mg, 91.1%) was synthesized from **30** (300 mg, 2 mmol) according to the procedure used to prepare **9**, obtained as white solids.^{1}H-NMR (400 MHz, Acetone-d_6) δ 12.42 (1H, s, 2-OH), 7.58 (1H, dd, *J* = 8.1, 1.5 Hz, H-4), 7.31 (1H, m, H-6), 6.84 (1H, m H-5), 5.16 (2H, s, -OCH_2O-), 3.44 (3H, s, -OCH_3), 2.63 (3H, s, -CH_3). MS (ESI) *m/z:* 197.1 $(M + H)^+$. The spectroscopic data of **31** were consistent with the reference. [S5]

(*E*)-3-(2,2-diphenylbenzo[*d*][1,3]dioxol-5-yl)-1-(2-hydroxy-3-(methoxymethoxy)phenyl)prop-2-en-1-one, 32

Compound **32** (161 mg, 56.1%) was synthesized from **31** (110 mg, 0.6 mmol) and **2,2-diphenylbenzo[*d*][1,3]dioxole-5-carbaldehyde** (180mg, 0.6mmol) according to the procedure used to prepare **11a**, obtained as yellow oil. ^{1}H-NMR (400 MHz, $CDCl_3$) δ 7.88 – 7.84 (4H, m, H-2′, H-3, H-7 and H-5′), 7.68 (1H, t, *J* = 7.6 Hz, H-6), 7.61 – 7.57 (4H, m, H-*o* of diphenylmethane), 7.48 – 7.41 (6H, m, H-*m, p* of diphenylmethane), 7.37 (1H, dd, *J* = 8.1, 1.7 Hz, H-6′), 7.29 (1H, dd, *J* = 9.0, 2.9 Hz, H-5), 7.05 (1H, d, *J* = 8.1 Hz, -<u>CH</u>=CHCO-), 6.90 (1H, d, *J* = 9.0 Hz, -CH=<u>CH</u>CO-), 5.17 (2H, s, -OCH_2O-), 3.43 (3H, s, -CH_3). MS (ESI) *m/z:* 481.1 $(M + H)^+$

2-(2,2-diphenylbenzo[d][1,3]dioxol-5-yl)-8-(methoxymethoxy)chroman-4-one, 33

Compound **33** (20 mg, 42.3%) was synthesized from **32** (40 mg, 0.1 mmol) according to the procedure used to prepare **12a**, obtained as yellow white. ^{1}H-NMR (400 MHz, Acetone-d_6) δ 7.61 – 7.57 (4H, m, H-*o* of diphenylmethane), 7.46 – 7.38 (7H, m, H-*m, p* of diphenylmethane and H-7), 7.23 (1H, t, *J* = 9.0, H-6), 7.20 (1H, d, *J* = 1.8 Hz, H-5′), 7.07 – 7.04 (1H, m, H-6′), 6.99 (1H, m, H-5), 6.97 (1H, s, H-2′), 5.46 (1H, dd, *J* = 13.2, 2.8 Hz, H-2), 5.15 (2H, s, -OCH_2O-), 3.40 (3H, s, -CH_3), 3.12 (1H, dd, *J* = 16.8, 13.2 Hz, H-3a), 2.76 (1H, dd, *J* = 16.8, 2.9 Hz, H-3b). MS (ESI) *m/z:* 481.1 $(M + H)^+$

2-(2,2-diphenylbenzo[*d*][1,3]dioxol-5-yl)-8-hydroxy-4*H*-chromen-4-one, 34

Compound **34** (213 mg, 77.9%) was synthesized from **33** (300 mg, 0.63 mmol) according to the procedure used to prepare **13a**, obtained as yellow white. ^{1}H-NMR (500 MHz, DMSO-d_6) δ 10.46 (1H, s, 8-OH), 7.87 (1H, s, H-2′), 7.81 (1H, d, *J* = 8.3 Hz, H-5′), 7.61-7.60 (4H, m, H-*o* of diphenylmethane), 7.51-7.49 (7H, m, H-*m,p* of diphenylmethane and H-7), 7.29 (3H, m, H-6′, H-5 and H-6), 6.97 (1H, s, H-3). MS (ESI) *m/z:* 425.1 $(M + H)^+$

2-(2,2-diphenylbenzo[*d*][1,3]dioxol-5-yl)-8-(2-hydroxyethoxy)-4*H*-chromen-4-one, 35

Compound **35** (70 mg, 30.6%) was synthesized from **34** (213 mg, 0.49 mmol) according to the procedure used to prepare **16a**. The crude product was purified by column chromatography

(PE/DCM/THF = 20:20:3) to give **35** as light yellow solids. ^{1}H-NMR (400 MHz, Acetone-*d*$_6$) δ 7.74 (1H, dd, *J* = 8.3, 1.8 Hz, H-5′), 7.68 (1H, d, *J* = 1.8 Hz, H-2′), 7.65 – 7.57 (5H, m, H-*o* of diphenylmethane and H-6′), 7.49 – 7.39 (7H, m, H-*m,p* of diphenylmethane and H-7), 7.32 (1H, t, *J* = 8.0 Hz, H-6), 7.14 (1H, d, *J* = 8.3 Hz, H-5), 6.76 (1H, s, H-3), 4.30 (2H, m, -OCH$_2$-), 3.99 (2H, m, -OCH$_2$-). MS (ESI) *m*/*z:* 479.1 (M + H)$^+$

2-((2-(2,2-diphenylbenzo[*d*][1,3]dioxol-5-yl)-4-oxo-4*H*-chromen-8-yl)oxy)ethyl (E)-3-(4-acetoxyphenyl)acrylate, 36

Compound **36** (46 mg, 46.3%) was synthesized from **35** (70 mg, 0.15 mmol) according to the procedure used to prepare **17a**. The crude product was purified by column chromatography (PE/acetone = 2:1) to give **36** as yellow solids. ^{1}H-NMR (400 MHz, Acetone-*d*$_6$) δ 7.69 - 7.63 (2H, m, -<u>CH</u>=CHCO- and H-5′), 7.57 – 7.53 (3H, m, H-2″,6″ and H-2′), 7.65 – 7.57 (5H, m, H-*o* of diphenylmethane and H-6′), 7.49 – 7.39 (7H, m, H-*m,p* of diphenylmethane and H-7), 7.38 (1H, t, *J* = 8.0 Hz, H-6), 7.15 (1H, d, *J* = 8.3 Hz, H-5), 7.02 (2H, d, *J* = 8.6 Hz, H-3″,5″), 6.93 (1H, s, H-3), 6.63 (1H, d, *J* = 16.0 Hz, -CH=<u>CH</u>CO-), 4.64 (2H, m, -OCH$_2$-), 4.45 (2H, m, -OCH$_2$-), 2.11 (3H, s, -CH$_3$). MS (ESI) *m*/*z:* 667.2 (M + H)$^+$

2-((2-(3,4-dihydroxyphenyl)-4-oxo-4*H*-chromen-8-yl)oxy)ethyl-(*E*)-3-(4-hydroxyphenyl)acrylate, 4l

Compound **4l** (6.1 mg, 46.3%) was synthesized from **36** (17 mg, 0.03 mmol) according to the procedure used to prepare 6. The crude product was purified by column chromatography (PE/acetone = 2:1) to give **4l** as yellow solids. ^{1}H-NMR (400 MHz, DMSO-*d*$_6$) δ 10.01 (1H, s, 3′-OH), 9.89 (1H, s, 4′-OH), 9.38 (1H, s, 4″-OH), 7.58 (1H, dd, *J* = 8.1 Hz, 1.5 Hz, H-5′), 7.57 (1H, d, *J* = 16.0 Hz, -<u>CH</u>=CHCO-), 7.51 (1H, dd, *J* = 8.1 Hz, 1.5 Hz, H-6′), 7.48 (2H, d, *J* = 8.7 Hz, H-2″,6″), 7.45(1H, dd, *J* = 7.2 Hz, 2.3 Hz, H-7), 7.44 (1H, s, H-2′), 7.38 (1H, t, *J* = 8.0 Hz, H-6), 6.89 (1H, d, *J* = 8.8 Hz, H-5), 6.76 (2H, d, *J* = 8.7 Hz, H-3″,5″), 6.74 (1H, s, H-3), 6.42 (1H, d, *J* = 15.9 Hz, -CH=<u>CH</u>CO-), 4.62 (2H, m, -OCH$_2$-), 4.49 (2H, m, -OCH$_2$-). ^{13}C-NMR (151 MHz, DMSO-*d*$_6$) δ 177.19, 166.96, 166.29, 163.12, 160.29, 159.26, 149.93, 148.07, 148.00, 146.40, 146.13, 145.63, 144.30, 133.01, 130.76, 130.07, 125.79, 125.41, 125.38, 124.82, 122.35, 119.10, 117.48, 116.47, 116.33, 116.17, 115.31, 115.25, 114.14, 113.81, 105.03, 67.45, 62.55. HR-MS (ESI) calcd for $C_{26}H_{20}O_8$: 460.11582, found [M + H]$^+$: 461.12222.

1.2. Synthesis of ***Series II*** *compounds*

1.2.1. Synthesis of compound **5g**

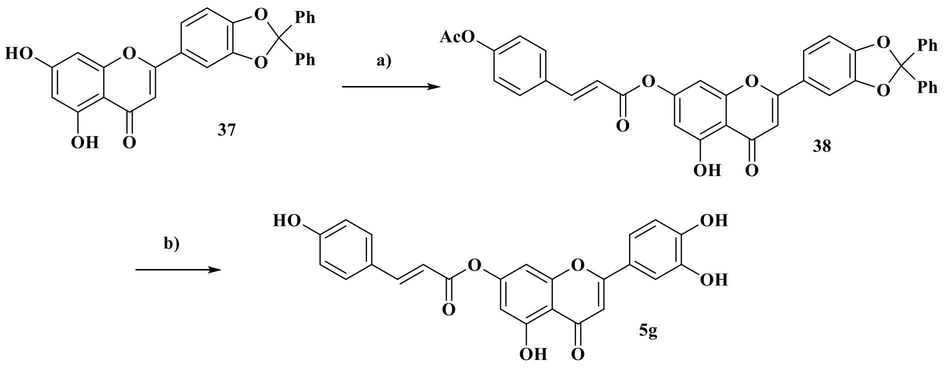

a, 4-acetylcoumaric acid, $SOCl_2$, DMF, DCE, 80°C, 2h; b, CH_3COOH/HCl, Acetone, r.t. 24h.

Scheme S5. Synthesis of compound 5g.

2-(2,2-diphenylbenzo[*d*][1,3]dioxol-5-yl)-5-hydroxy-4-oxo-4*H*-chromen-7-yl (*E*)-3-(4-acetoxyphenyl)acrylate, 38

Compound **38** (32 mg, 72.0%) was synthesized from **37** (32mg, 0.07mmol) and 4-acetoxycinnamic acid (12.4mg, 0.07 mmol) according to the procedure used to prepare **15a** to give **38** as white solids. ^{1}H-NMR (400MHz, $CDCl_3$) δ 7.70 (1H, d, *J* = 16.0Hz, -<u>CH</u>=CHCO-), 7.56-7.63 (4H, m, H-*o* of

diphenylmethane), 7.53 (2H, d, *J*= 8.4Hz, H-4″, H-6″), 7.45 (1H, dd, *J* = 8.0, 1.6Hz, H-5′), 7.35-7.43 (7H, m, H-*m, p* of diphenylmethane ,H-2′), 7.11 (2H, d, *J* = 8.8Hz, H-3″, H-5″), 6.99 (1H, d, *J* = 8.0Hz, H-6′), 6.52 (1H, s, H-3), 6.50 (2H, d, *J* = 2.0Hz, H-8), 6.43 (1H, d, J = 16.0Hz, -CH=CHCO-), 6.40 (1H, d, *J* = 2.0Hz, H-6), 2.30 (3H, s, CH_3). MS (ESI) *m*/*z:* 639.2 $(M + H)^+$

(*E*)-2-(3,4-dihydroxyphenyl)-5-hydroxy-4-oxo-4*H*-chromen-7-yl 3-(4-hydroxyphenyl)acrylate, 5g

Compound **5g** (22 mg, 27.1%) was synthesized from **38** (32mg, 0.05mmol) according to the procedure used to prepare **4k**. The crude product was purified by column chromatography (DCM/MeOH = 50:1) to give **5g** as yellow solids. ^{1}H-NMR (400 MHz, Acetone-*d*$_6$) δ 7.92 (2H, d, *J* = 7.4 Hz, H-2″, 6″), 7.53 (1H, s, H-2′), 7.48 (1H, d, H-6′), 7.01 (1H, d, *J* = 7.5 Hz, H-5′), 6.91 (2H, d, *J* = 8.5 Hz, H-3″, 5″), 6.77 (1H, s, H-3), 6.62 (1H, s, H-8), 6.40 (1H, s, H-6), 4.66 (2H, s, -OCH_2-), 4.54 (2H, s, -OCH_2-). ^{13}C-NMR (150 MHz, DMSO-*d*$_6$) δ 182.67, 165.36, 164.92, 161.21, 160.94, 156.56, 156.27, 150.57, 148.06, 146.23, 133.13, 131.38, 130.03, 129.00, 125.25, 121.55, 119.80, 116.45, 116.34, 114.11, 112.88, 108.35, 105.64, 103.85, 101.84. HR-MS (ESI) calcd for $C_{24}H_{16}O_8$: 432.08452, found$[M + H]^+$: 433.09149.

1.3. Synthesis of ***Series Ⅲ*** *compounds*

1.3.1. Synthesis of compound **6**

Scheme S6. Synthesis of compound 6

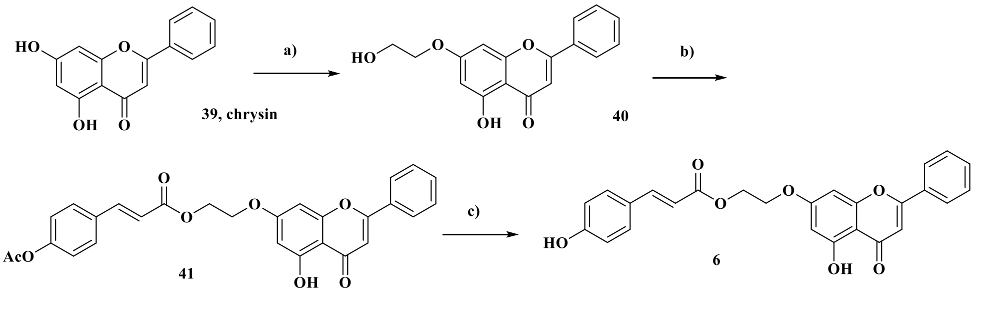

a, 6, $TsOCH_2CH_2OH,K_2CO_3$, MeCN, 80°C, 12h;b, 4-acetylcoumaric acid, EDCI, DMAP, TEA,THF, r.t. 24h; c, CH_3COOH/HCl, Acetone, r.t. 24h.

Scheme S6. Synthesis of compound 6.

5-hydroxy-7-(2-hydroxyethoxy)-2-phenyl-4*H*-chromen-4-one, 40

Compound **40** (223 mg, 37.1%) was synthesized from **39** (508 mg, 2 mmol) according to the procedure used to prepare **16a**. The crude product was purified by column chromatography (DCM/Acetone = 10:1) to give **40** as white solids. ^{1}H-NMR (400 MHz, DMSO-*d*$_6$) δ 8.07 (2H, d, *J* = 7.1 Hz, H-3″, 5″), 7.70 (1H, d, *J* = 8.2 Hz, H-6′), 7.59 – 7.56 (2H, m, H-2″, 6″), 7.62 – 7.54 (3H, m, H-3′, 4′, 5′), 7.44 (1H, d, *J* = 8.1 Hz, H-2′), 7.01 (1H, s, H-3), 6.82 (1H, d, *J* = 1.8 Hz, H-8), 6.39 (1H, d, *J* = 1.8 Hz, H-6), 4.34 (2H, s, -OCH_2-), 4.32 (2H, s, -OCH_2-). MS (ESI) *m*/*z:* 299.1 $(M + H)^+$. The spectroscopic data of **40** were consistent with the reference. [S6]

2-((5-hydroxy-4-oxo-2-phenyl-4*H*-chromen-7-yl)oxy)ethyl (*E*)-3-(4-acetoxyphenyl)acrylate, 41

Compound **41** (170 mg, 89.3%) was synthesized from **40** (223mg, 0.75mmol) and 4-acetoxycinnamic acid (464 mg, 2.25mmol) according to the procedure used to prepare **17a** to give **41** as white solids. ^{1}H-NMR (400 MHz, DMSO-*d*$_6$) δ 12.92 (1H, s, 5-OH), 8.02 (2H, d, *J* = 7.1 Hz, H-3″ , 5″), 7.73 (1H, d, *J* = 8.2 Hz, H-6′), 7.62 – 7.54 (3H, m, H-3′,4′,5′), 7.46 (1H, d, *J* = 8.1 Hz, H-2′), 7.17 （1H, d, *J* = 8.1 Hz, H-2″, 6″）7.03 (1H, s, H-3), 6.83 (1H, d, *J* = 1.8 Hz, H-8), 6.39 (1H, d, *J* = 1.8 Hz, H-6), 4.37 (2H, s, -OCH_2-), 4.36 (2H, s, -OCH_2-), 2.45 (3H, s, CH_3). MS (ESI) *m*/*z:* 487.1 $(M + H)^+$

(*E*)-2-((5-hydroxy-4-oxo-2-phenyl-4*H*-chromen-7-yl)oxy)ethyl 3-(4-hydroxyphenyl)acrylate, 6

Compound **6** (41 mg, 26.8%) was synthesized from **41** (170 mg, 0.67 mmol) according to the procedure used to prepare **4k**. The crude product was purified by column chromatography (PE/acetone = 3:1) to give **6** as white solids. ^{1}H-NMR (400 MHz, DMSO-d_6) δ 12.93(1H, s, 5-OH), 10.0 (1H, s, 4''-OH), 8.05(2H, d, J = 7.1 Hz, H-3'' , 5''), 7.70 (1H, d, J = 8.2 Hz, H-6'), 7.62 – 7.54 (3H, m, H-3',4',5'), 7.44 (1H, d, J = 8.1 Hz, H-2'), 7.15（1H, d, J = 8.1 Hz, H-2'', 6''）7.01 (1H, s, H-3), 6.82 (1H, d, J = 1.8 Hz, H-8), 6.39 (1H, d, J = 1.8 Hz, H-6), 4.34 (2H, s, -OCH$_2$-), 4.32 (2H, s, -OCH$_2$-). ^{13}C-NMR (100 MHz, DMSO-d_6) δ 167.19, 161.91, 158.02, 145.97, 143.55, 132.84 (2), 131.09 (2), 129.81, 127.13, 116.43 (2), 114.35 (2), 111.33, 106.06, 99.28, 98.60, 94.09, 67.50, 62.83. HR-MS (ESI) calcd for $C_{24}H_{18}O_7$: 418.10525, found [M + H]$^+$: 419.10544.

1.3.2. Synthesis of compound **8**

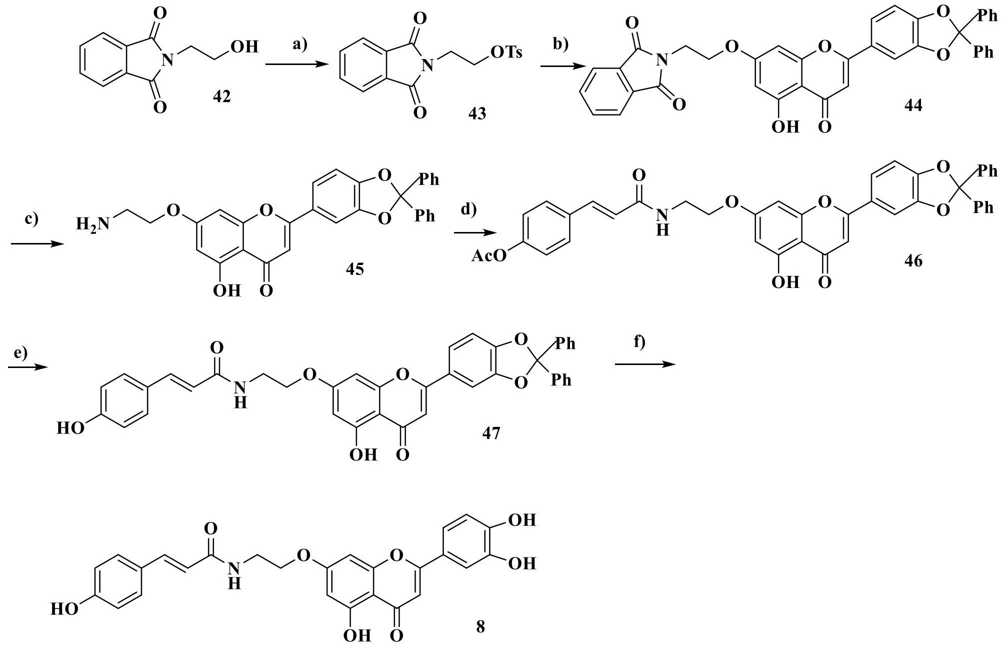

a),TsCl, TEA, DCM, 40°C, 24h; b) 45, K_2CO_3, MeCN, 80°C, 13h; c) CH_3NH_2 in EtOH, THF, r.t. 12h; d) 4-acetylcoumaric acid, EDCI, DMAP, TEA, THF, r.t. 40°C, 12h; e) K_2CO_3 in MeOH, DCM, r.t. 24h; f) CH_3COOH/HCl, acetone, r.t.12h

Scheme S7. Synthesis of compound 8.

2-(1,3-dioxoisoindolin-2-yl)ethyl 4-methylbenzenesulfonate, 43

N-(2-hydroxyethyl)phthalimide **42** (191 mg, 1 mmol) was dissolved in 5 mL dry CH_2Cl_2, and then TsCl (229 mg, 1.2 mmol) and TEA(278uL, 2 mmol) were added into the mixture. The reaction mixture was stirred 16 h at 40℃. The resulting mixture was diluted with CH_2Cl_2（10mL）, washed with saturated aqueous $NaHCO_3$ and NaCl successively, and dried over anhydrous Na_2SO_4. After concentrated *in vacuo*, the residue was purified by column chromatography (PE/acetone = 5:1) to give **43** (254mg, 73.6%) as white solid. ^{1}H-NMR (400 MHz, $CDCl_3$) δ 7.81 (2H, m, H-2,6), 7.73 (2H, m, H-3,4), 7.69 (1H, d, J = 8.2 Hz, H-2', 6'), 7.16 (2H, d, J = 8.2 Hz, H-3', 5'), 4.32 (2H, t, J = 5.3 Hz, -OCH$_2$-), 3.93 (2H, t, J = 5.4 Hz, -OCH$_2$-). MS (ESI) m/z: 346.1(M + H)$^+$. The spectroscopic data of **31** were consistent with the reference. [S7]

2-(2-((5-hydroxy-2-(2-methyl-2-phenylbenzo[*d*][1,3]dioxol-5-yl)-4-oxo-4*H*-chromen-7-yl)oxy)ethyl) isoindoline-1,3-dione, 44

Compound **37** (51 mg, 0.11 mmol) was dissolved in MeCN (5 mL), and then **43** (48 mg, 0.14 mmol) and K_2CO_3 (23 mg, 0.16 mmol) were added into the mixture. After stirring at 80℃ for 12h, water (10 mL) was added and the mixture was extracted by ethyl acetate. The organic phase was washed with saturated aqueous NaCl, and dried over anhydrous Na_2SO_4. After concentrated *in vacuo*, the residue was purified by column chromatography (PE/DCM/acetone = 20:20:1) to give **44** (42 mg, 59.8%) as

yellow solid. ^{1}H-NMR (400 MHz, $CDCl_3$) δ 7.87 (2H, dd, *J* = 5.3, 3.1 Hz, H-2″, 6″), 7.73 (2H, dd, *J* = 5.4, 3.1 Hz, H-3″, 4″), 7.59-7.57 (4H, m, H-*o* of diphenylmethane), 7.44 (1H, d, *J* = 8.3 Hz, H-6′), 7.41-7.39 (6H, m, H-*m, p* of diphenylmethane), 7.37 (1H, s, H-2′),c 6.98 (1H, d, *J* = 8.3 Hz, H-5′), 6.51 (1H, s, H-3), 6.45 (1H, d, *J* = 2.0 Hz, H-8), 6.32 (1H, d, *J* = 2.0 Hz, H-6), 4.30 (2H, t, *J* = 5.7 Hz, $-OCH_2-$), 4.14 (2H, *J* = 5.6 Hz, $-OCH_2-$). MS (ESI) *m/z:* 522.2(M + H)$^+$

(*E*)-4-(3-((2-((5-hydroxy-2-(2-methyl-2-phenylbenzo[*d*][1,3]dioxol-5-yl)-4-oxo-4*H*-chromen-7-yl)oxy)ethyl)amino)-3-oxoprop-1-en-1-yl)phenyl acetate, 46

Compound **44** (15 mg, 0.024 mmol) was dissolved in 1 mL dry THF, and then methylamine alcohol solution (1mL) was added. After stirring at 40℃ for 2h, saturated aqueous NaCl solution (5 mL) was added and the mixture was extracted by DCM. The organic phase was dried over anhydrous Na_2SO_4 and concentrated *in vacuo* to give crude **45**. Compound **46** (12 mg, 72.5%) was synthesized from **45** (dissolved in dry THF) according to the procedure used to prepare **17a**. The crude product was purified by column chromatography (DCM/methanol = 50:1) to give **60** as yellow solids. ^{1}H-NMR (500 MHz, DMSO-*d*$_6$) δ 12.87 (1H, s, 5-OH), 8.44 (1H, s, -NH), 7.85 (1H, s, H-2′), 7.78 (1H, d, *J* = 8.2 Hz, H-6′), 7.63 (2H, d, *J* = 8.4 Hz, H-2″, 6″), 7.59 (4H, m, H-*o* of diphenylmethane), 7.49 (6H, m, H-*p, m* of diphenylmethane), 7.28 (1H, d, *J* = 8.3 Hz, H-5′), 7.20 (2H, d, *J* = 8.5 Hz, H-3″, 5″), 6.99 (1H, s, H-3), 6.91 (1H, d, *J* = 1.7 Hz, H-8), 6.68 (1H, d, *J* = 15.8 Hz, -CH=CHCO-), 6.44 (1H, d, *J* = 1.7Hz, H-6), 4.23 (2H, t, *J* = 5.0 Hz, $-OCH_2-$), 3.63 (2H, t, *J* = 5.2 Hz, $-OCH_2-$).2.24(3H, s, $-CH_3$). MS (ESI) *m/z:* 620.2(M + H)$^+$

(*E*)-*N*-(2-((5-hydroxy-2-(2-methyl-2-phenylbenzo[*d*][1,3]dioxol-5-yl)-4-oxo-4H-chromen-7-yl)oxy)ethyl)-3-(4-hydroxyphenyl)acrylamide, 47

Compound **61** (18 mg, 62.6%) was synthesized from **60** according to the procedure used to prepare **28**. The crude product was purified by column chromatography (PE/acetone = 2:1) to give **61** as yellow solids. ^{1}H-NMR (500 MHz, DMSO-*d*$_6$) δ 12.87 (1H, s, 5-OH), 8.47 (1H, s, -NH), 7.85 (1H, s, H-2′), 7.78 (1H, d, *J* = 8.2 Hz, H-6′), 7.63 (2H, d, *J* = 8.3 Hz, H-2″, 6″), 7.59 (4H, m, H-*o* of diphenylmethane), 7.49 (6H, m, H-*p, m* of diphenylmethane), 7.24 (1H, d, *J* = 8.3 Hz, H-5′), 7.20 (2H, d, *J* = 8.2 Hz, H-3″, 5″), 6.99 (1H, s, H-3), 6.91 (1H, d, *J* = 1.7 Hz, H-8), 6.68 (1H, d, *J* = 15.8 Hz, -CH=CHCO-), 6.45 (1H, d, *J* = 1.7Hz, H-6), 4.23 (2H, t, *J* = 5.0 Hz, $-OCH_2-$), 3.63 (2H, t, *J* = 5.2 Hz, $-OCH_2-$). MS (ESI) *m/z:* 578.2(M + H)$^+$

(*E*)-*N*-(2-((2-(3,4-dihydroxyphenyl)-5-hydroxy-4-oxo-4*H*-chromen-7-yl)oxy)ethyl)-3-(4-hydroxyphenyl)acrylamide, 8

Compound **8** (2.4 mg, 32.1%) was synthesized from **47** according to the procedure used to prepare **4k**. The crude product was washed by diethyl ether to give **8** as light-yellow solids. ^{1}H-NMR (500 MHz, DMSO-*d*$_6$) δ 13.00 (1H, s, 5-OH), 10.01 (1H, s, 4″-OH), 9.85 (1H, s, 3″-OH), 9.39 (1H, s, 4″-OH), 8.29(1H, s, -NH), 7.85 (1H, s, H-2′), 7.78 (1H, d, *J* = 8.2 Hz, H-6′), 7.63 (2H, d, *J* = 8.3 Hz, H-2″, 6″), 7.24 (1H, d, *J* = 8.3 Hz, H-5′), 7.20 (2H, d, *J* = 8.2 Hz, H-3″, 5″), 6.99 (1H, s, H-3), 6.91 (1H, s, H-8), 6.76 (s, 1H), 6.49 (1H, d, *J* = 15.7 Hz, -CH=CHCO-), 6.42 (1H, s, H-6), 4.22 (2H, t, *J* = 5.0 Hz, $-OCH_2-$), 3.60 (2H, d, *J* = 4.9 Hz, $-OCH_2-$). ^{13}C-NMR (150 MHz, DMSO-*d*$_6$) δ 182.23, 166.21, 164.70, 164.63, 161.62, 159.31, 157.64, 150.28, 146.19, 139.51, 129.69, 126.25, 121.83, 119.53, 118.73, 116.37, 116.17, 114.00, 105.18, 103.51, 98.85, 93.47, 67.71, 63.52. HR-MS (ESI) calcd for $C_{26}H_{21}NO_8$: 475.12672, found [M + H]$^+$: 476.13400.

2. Inhibition of Aβ production in APP overexpressed cells

HEK293 cells stably overexpressing human APP695 were plated in a poly-D-lysine-coated 96-well plates and grown to a density of 80-90%. Compounds were dissolved in DMSO to make a 10 mM stock solution and diluted in DMEM complete medium to the final concentrations: 0.1, 1.0, 5.0, 10.0, 25.0 and 50.0 μM. DMSO was used at a 1:200 dilution as a negative control and a commercially available BACE1 inhibitor (C3, also known as BACE inhibitor IV) as a positive control. After 23 h of incubation the cell supernatants were directly used for measurement in the V-Plex Aβ Peptide Panel

1 (6E10) Kit from Meso Scale Diagnostics (Rockville, Maryland) for measuring Aβ38, -40 and -42 simultaneously. Cells were washed with PBS and lysed in Triton X100 lysis buffer for subsequent protein concentration (BCA) measurement.

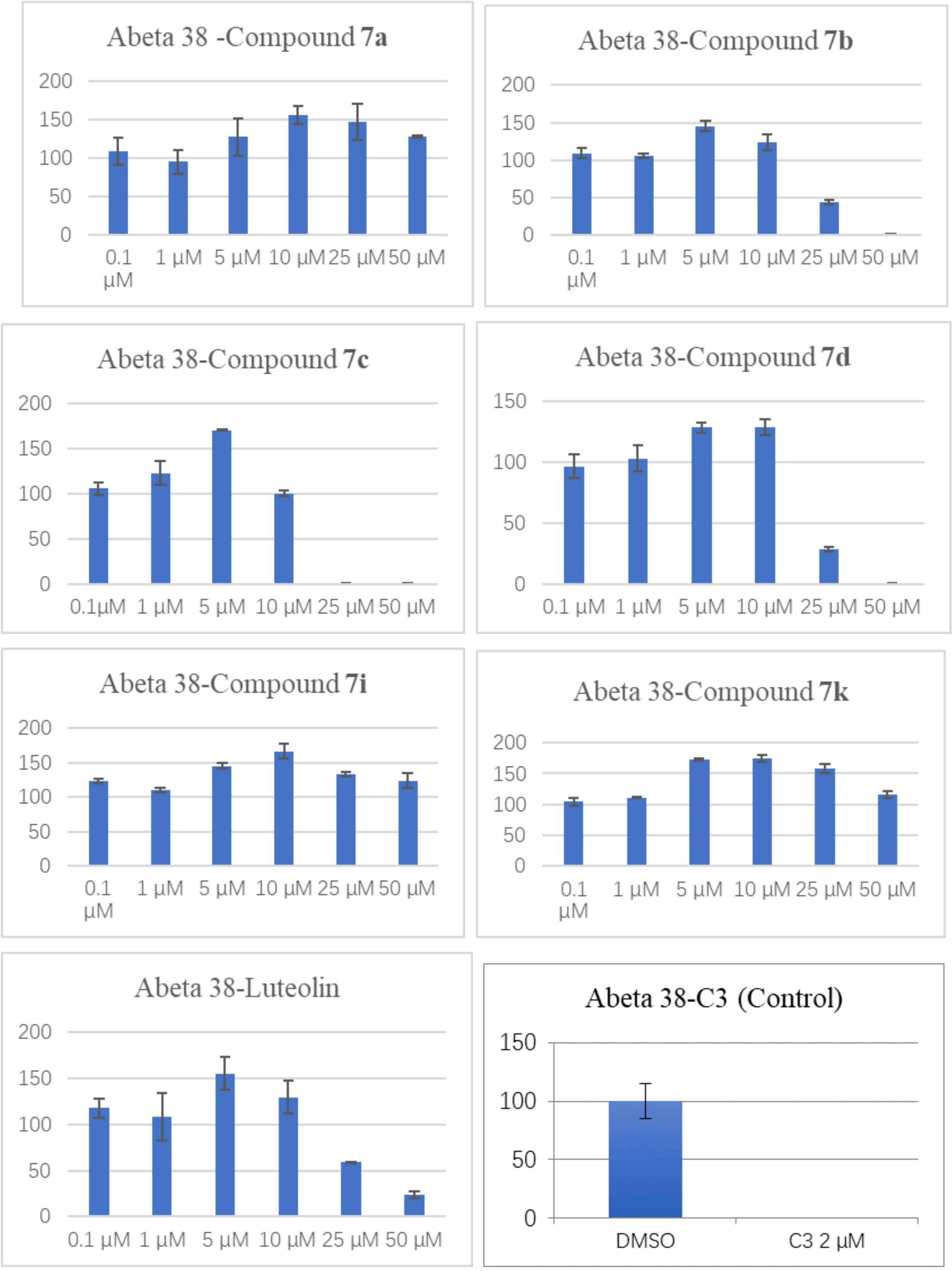

Figure 1. The normalized Aβ38 level in APP overexpressing HEK293 cells after the treatment of **7a**–**7d**, **7i**, **7k** and **luteolin** compared with DMSO (100%) the known inhibitor C3.

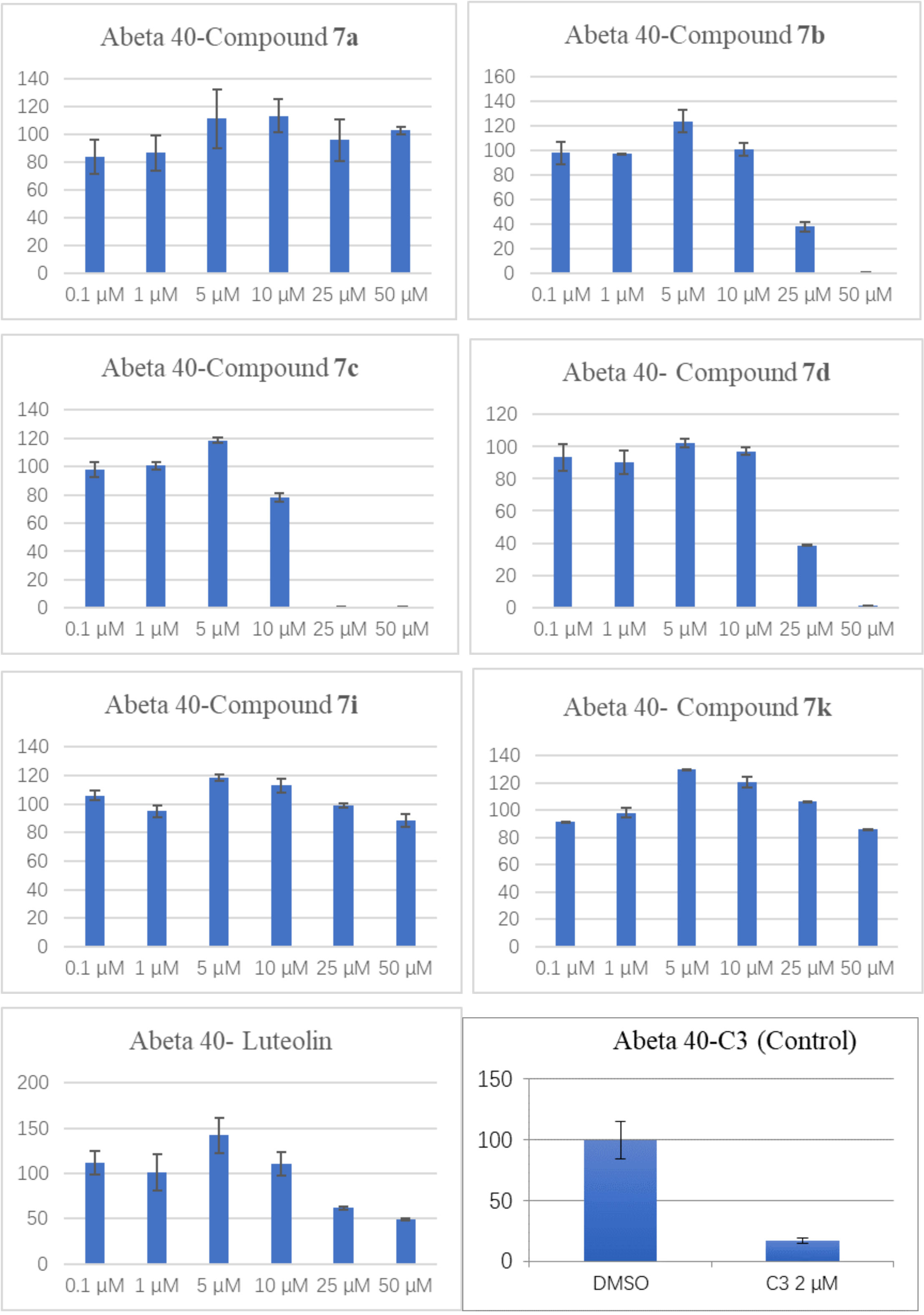

Figure 2. The normalized Aβ38 level in APP overexpressing HEK293 cells after the treatment of **7a**–**7d**, **7i**, **7k** and **luteolin** compared with DMSO (100%) the known inhibitor C3.

3. Kinetic analysis

The Dixon plot is a graphical method [plot of 1/enzyme velocity (1/V) against inhibitor concentration (I)] for determination of the type of enzyme inhibition and was used to determine the dissociation or inhibition constant (Ki) for the enzyme inhibitor complex (Cornish-Bowden, 1974). The Dixon plots for BACE1 inhibition were obtained in the presence of various concentrations of

BACE1 substrate (250, 500, 750 nM) and the concentrations of **4l, 7c** and **7k** as follows: 0.1μM, 0.5μM, 1.0μM. In this way, the inhibition constants (Ki) of **4e** were determined by interpretation of Dixon plots, where the value of the X-axis represents -Ki when 1/V=0.

Compound **4l** and **7k** showed mix-competitive mode, and the Ki were 0.80μM and 0.32μM.

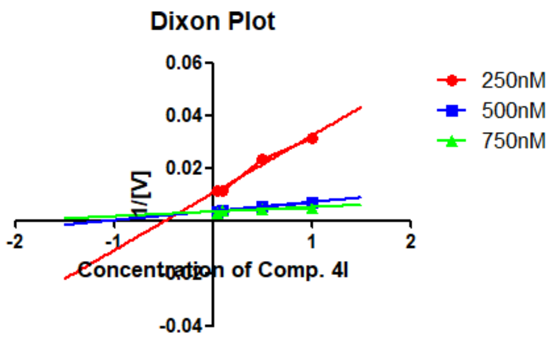

Figure 3. Kinetic analysis of **4l.**

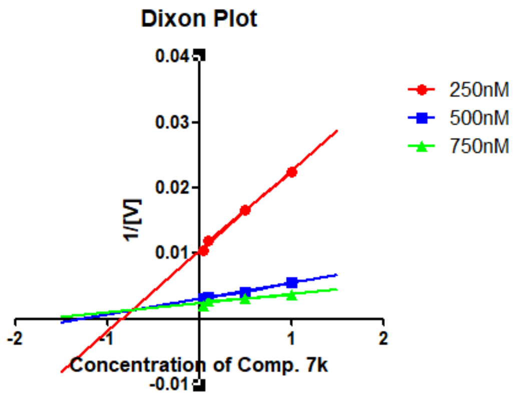

Figure 4. Kinetic analysis of **7k.**

4. S-curve for IC_{50} calculation

The BACE1 FRET assay kit was purchased from the PanVera Co. (Invitroge, USA). The assay was carried out according to the supplied manual with modifications. Briefly, assays were performed in triplicate in 384-well black plates with a mixture of 5 μL of BACE1 (1.0 U/ml), 5 μL of the substrate (750 nM, Rh-EVNLDAEFK- Quencherin 50 mM, ammonium bicarbonate), and 5 μL of compound dissolved in 10% DMSO. The fluorescence intensity was measured with a TECAN infinite 200 microplate reader for 60 min at 25 °C in the dark. The mixture was irradiated at 544 nm and the emission intensity recorded at 590 nm. The percent inhibition (%) was obtained by the following equation: Inhibition % = (1-SS/SC) × 100%, where SC is the slope of fluorescence change of the control (enzyme, buffer, and substrate) during 60 min, and SS is the slope of fluorescence change of the tested samples (enzyme, sample solution, and substrate) during 60 min of measurement. IC_{50} values were calculated from the nonlinear curve fitting of percentage inhibition against inhibitor concentration using Prism 3.0 software.

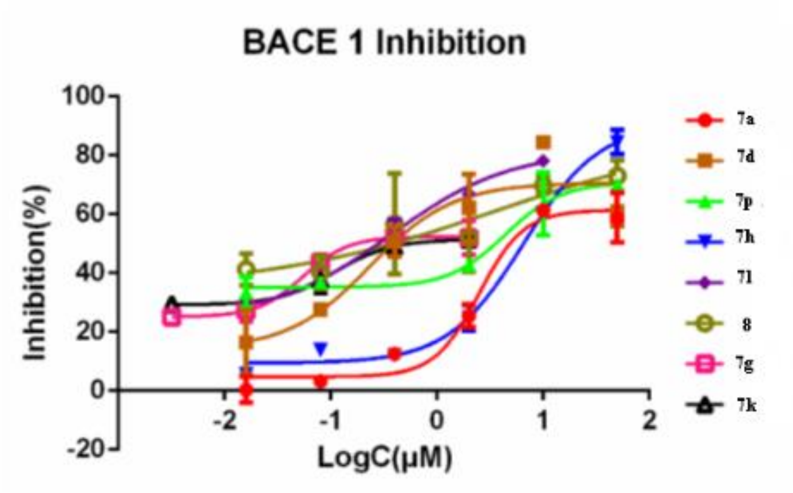

Figure 5. S-curve for IC50 calculation of Compound **7a, 7d, 7p, 7h, 7g, 7k** and **8.**

5. ClogP and BBB level calculation

The LogP and BBB level calculation were operated on the ADMETlab (http://admet.scbdd.com/), an open and free platform for druglikeness analysis and systematic ADMET evaluation. The LogP calculation is from RDKit directly. The BBB penetration calculation is done by support vector machines (SVM) method and ECFP2 as fingerprint (the model based on SVM and ECFP2 was the optimal one based on the choice from 20 classification models by five folds cross validation and external validation dataset). [S8]

In the practical use of the platform, the compounds chosen by us were transformed into SMILES form and input on the web. Then the LogP and BBB level calculation was done after our choosing the target function and submitting the compound(s). The LogP was output just as a clogP value. The BBB penetration results were output a Category number (0 or 1) and a Probability value. If the probability value >0.5, the category was label as 1, otherwise as 0. The value of 1 represents BBB+ (BBB penetration with high probability), whereas 0 represents BBB- (low probability). The results of compounds **1,3, 4l, 7a, 7c, 7k** and **7l** were exhibited in Table S2.

Table 2. ClogP and BBB penetration .

Compound	clogP	Category	Probability	BBB penetration probability
Luteolin	2.68	0	0.464	BBB-
**3**	3.92	0	0.403	BBB-
**4l**	4.21	1	0.659	BBB+
**7a**	4.21	1	0.653	BBB+
**7c**	3.92	0	0.5	BBB-
**7k**	3.61	1	0.658	BBB+
**7l**	3.81	1	0.521	BBB+

References

S1. Chen, L.C.; Hsu, K.C.; Chiou, L.C.; Tseng, H.J.; Huang, W.J. Total synthesis and metabolic stability of hispidulin and its d-labelled derivative. *Molecules*, **2017**, *22*, 1-14.

S2. Lee, Y.R.; Li, X.; Kim, J.H. Concise Total Synthesis of Biologically Interesting Mallotophilippens C and E. *J. Org. Chem.* **2008**, *73*, 4313-4316.

S3. Su, Z.R.; Fan, S.Y.; Shi, W.G.; Zhong, B.H. Discovery of xanthine oxidase inhibitors and/or α-glucosidase inhibitors by carboxyalkyl derivatization based on the flavonoid of apigenin. *Bioorg. Med. Chem. Lett*. **2015**, *14*, 2778-2781.

S4. Saito, Y.; Mizokami, A.; Tsurimoto, H.; Izumi, K.; Goto, M.; Nakagawa-Goto, K. 5'-Chloro-2,2'-dihydroxychalcone and related flavanoids as treatments for prostate cancer. *Eur. J. Med. Chem*.**2018**, *157*, 1143-1152.

S5. Zhang, G.N.; Huan, Y.; Wang, X.; Sun, S.J.; Shen, Z.F.; Fang, W.S. Design, synthesis and metabolic regulation effect of farnesoid X receptor (FXR) antagonistic benzoxepin-5-ones. *Chin. Chem. Lett.*, **2017**, *7*, 1519-1522.

S6. De Groot, A.; Dommisse, R.; Lemiere, G. Selective cleavage of tert-butyldimethylsilylethers ortho to a carbonyl group by ultrasound. *Tetrahedron*, **2000**, *11*, 1541-1549.

S7. Laura, B.; Juan, R.; Andrea, O; Diego, G. Insights into the Pummerer synthesis of oxazolines. *Org. Biomol. Chem.*, **2016**, *36*, 8474-8485.

S8. Dong, J.; Wang, N.N.; Yao, Z.J.; Zhang, L.; Cheng, Y.; Ouyang, D.F.; Lu, A.P. ADMETlab: a platform for systematic ADMET evaluation based on a comprehensively collected ADMET database. J. Cheminform. **2018**, *10*, 29-40.
